# Microfluidic and Static Organotypic Culture Systems to Support *Ex Vivo* Spermatogenesis From Prepubertal Porcine Testicular Tissue: A Comparative Study

**DOI:** 10.3389/fphys.2022.884122

**Published:** 2022-06-02

**Authors:** Marc Kanbar, Francesca de Michele, Jonathan Poels, Stéphanie Van Loo, Maria Grazia Giudice, Tristan Gilet, Christine Wyns

**Affiliations:** ^1^ Andrology Lab, Institut de Recherche Expérimentale et Clinique (IREC), Université Catholique de Louvain, Brussels, Belgium; ^2^ Department of Gynecology-Andrology, Cliniques Universitaires Saint-Luc, Brussels, Belgium; ^3^ Microfluidics Lab, Department of Aerospace and Mechanical Engineering, University of Liege, Liege, Belgium

**Keywords:** spermatogonial stem cells, immature testicular tissue, organotypic culture, microfluidic culture, *in vitro* spermatogenesis, fertility preservation, cancer, boys

## Abstract

**Background:** *In vitro* maturation of immature testicular tissue (ITT) cryopreserved for fertility preservation is a promising fertility restoration strategy. Organotypic tissue culture proved successful in mice, leading to live births. In larger mammals, including humans, efficiently reproducing spermatogenesis *ex vivo* remains challenging. With advances in biomaterials technology, culture systems are becoming more complex to better mimic *in vivo* conditions. Along with improving culture media components, optimizing physical culture conditions (e.g., tissue perfusion, oxygen diffusion) also needs to be considered. Recent studies in mice showed that by using silicone-based hybrid culture systems, the efficiency of spermatogenesis can be improved. Such systems have not been reported for ITT of large mammals.

**Methods:** Four different organotypic tissue culture systems were compared: static i.e., polytetrafluoroethylene membrane inserts (OT), agarose gel (AG) and agarose gel with polydimethylsiloxane chamber (AGPC), and dynamic i.e., microfluidic (MF). OT served as control. Porcine ITT fragments were cultured over a 30-day period using a single culture medium. Analyses were performed at days (d) 0, 5, 10, 20 and 30. Seminiferous tubule (ST) integrity, diameters, and tissue core integrity were evaluated on histology. Immunohistochemistry was used to identify germ cells (PGP9.5, VASA, SYCP3, CREM), somatic cells (SOX9, INSL3) and proliferating cells (Ki67), and to assess oxidative stress (MDA) and apoptosis (C-Caspase3). Testosterone was measured in supernatants using ELISA.

**Results:** ITT fragments survived and grew in all systems. ST diameters, and Sertoli cell (SOX9) numbers increased, meiotic (SYCP3) and post-meiotic (CREM) germ cells were generated, and testosterone was secreted. When compared to control (OT), significantly larger STs (d10 through d30), better tissue core integrity (d5 through d20), higher numbers of undifferentiated spermatogonia (d30), meiotic and post-meiotic germ cells (SYCP3: d20 and 30, CREM: d20) were observed in the AGPC system. Apoptosis, lipid peroxidation (MDA), ST integrity, proliferating germ cell (Ki67/VASA) numbers, Leydig cell (INSL3) numbers and testosterone levels were not significantly different between systems.

**Conclusions:** Using a modified culture system (AGPC), germ cell survival and the efficiency of porcine germ cell differentiation were moderately improved *ex vivo*. We assume that further optimization can be obtained with concomitant modifications in culture media components.

## 1 Introduction

Cryopreservation of immature testicular tissue (ITT) containing spermatogonial stem cells (SSCs) is an ethically accepted and recommended approach (as per several oncology, pediatric and fertility societies recommendations) for fertility preservation (Practice Committee of the American Society for Reproductive Medicine. Electronic address, 2019; Mulder et al., 2021). It can be offered to prepubertal boys at risk of becoming infertile (e.g., following a gonadotoxic treatment for cancerous or benign diseases) with the aim of restoring their fertility if diagnosed with azoospermia when facing a child wish (Wyns et al., 2020).

Fertility preservation programs have been developed since the early 2000s, and to date, the most recent survey showed that more than a thousand boys have participated in Europe, Canada, and the United States (Goossens et al., 2020).

The largest series reporting follow-up data on the reproductive potential of these boys showed that 46% who had undergone a testicular biopsy for fertility preservation before the onset of spermatogenesis suffered from a severely impaired spermatogenesis (azoospermia in 29% of cases) after a median of 6.5 (2.6–14) years from gonadotoxic treatment completion. This makes the development of clinically-applicable fertility restoration methods using cryopreserved ITT an urgent matter in order to give these boys the hope to father their own biological child (Kanbar et al., 2021).

So far, three experimental methods have been considered for fertility restoration with cryopreserved ITT: tissue auto-grafting, SSCs transplantation, or *in vitro* maturation (IVM) of the tissue or the SSCs (Wyns et al., 2020). Autografting of frozen-thawed ITT has already led to the birth of a healthy female baby monkey using graft-derived sperm (Fayomi et al., 2019), while transplantation of SSCs led to offspring in some animal species and ICSI-mediated embryos in monkeys (Hermann et al., 2012). While both transplantation of ITT or SSCs back to the patient present the risk of cancer cell contamination of the tissue or cell suspension, and thus of disease relapse, IVM offers the advantage of circumventing this risk by using *in vitro* generated sperm to obtain embryos *ex vivo*.

Efforts to reproduce spermatogenesis *in vitro* have been ongoing since more than a 100 years (Wyns and Kanbar, 2022) and have been fueled in the past decade by advancements in *in vitro* culture techniques. Among the noteworthy achievements and developments were the creation of *in vitro* culture physical support systems (e.g., hanging drops, metallic wire grids, agarose blocks, meshed membranes, synthetic scaffolds), the development of enriched and complex culture media for metabolic support (e.g., using lymph/plasma clots, serum, growth factors and hormones, and more recently serum-free components) and attempts to the definition of most adequate culture conditions (e.g., sterility, temperature, humidity, gas content, pH, etc.) (Stukenborg et al., 2009; Richer et al., 2020; Wyns and Kanbar, 2022).

Yet, despite long-lasting efforts, IVM of prepubertal testicular tissue from rats (Reda et al., 2016; Matsumura et al., 2021; Saulnier et al., 2021) and larger mammals (Heckmann et al., 2020; Sharma et al., 2022) including humans (de Michele et al., 2017; Medrano et al., 2018; Portela et al., 2019a; Kurek et al., 2021) remained disappointing compared to achievements in mice where fertilization-competent spermatozoa leading to live births were produced (Sato et al., 2011; Yokonishi et al., 2014; Komeya et al., 2016) although the success of *in vitro* spermatogenesis in the latter species seemed to be strain-dependent (Portela et al., 2019b). In the best-case scenario for humans, few haploid germ cells were produced *in vitro* starting from SSCs found within a testicular cell suspension (Abofoul-Azab et al., 2018) or from intact ITT fragments of prepubertal boys (de Michele et al., 2018). Moreover, spermatogonia numbers decreased over the culture period.

Overcoming these limitations is therefore essential to allow for a potential clinical application of the technique in the future.

Since the early-2000s, tissue engineering technologies have been used to increasingly mimic the *in vivo* conditions notably in terms of tissue/cell perfusion and oxygenation amelioration (Lovett et al., 2009; Place et al., 2017) leading to an improved culture outcome (e.g., viability and function) in thick tissues like liver (Khong et al., 2007) and cardiac muscles (Vollert et al., 2014).

Recently, some of these technologies (bioreactors, microfluidic devices) have also been applied to the culture of testicular tissue fragments (Komeya et al., 2016; Yamanaka et al., 2018; Komeya et al., 2019; Matsumura et al., 2021), isolated seminiferous tubules (Perrard et al., 2016) and testicular cell suspensions (Dores et al., 2015; Baert et al., 2020) by different groups (reviewed by Oliver and Stukenborg, 2020).

The most exciting results in terms of *in vitro* spermatogenesis were recently reported by the group of Ogawa in Japan who implemented microfluidic (MF) systems. Using ITT fragments (0.5–6.5 mm^2^) from prepubertal mice, the group demonstrated the successful maturation and long-term (6 months) maintenance of the tissue *in vitro* using a polydimethylsiloxane (PDMS) based MF organotypic culture system (PDMS being a widely used and biocompatible silicone-based and gas-permeable organic polymer). Key findings were the maintenance of the endocrine function (until day 180) and the generation of fertilization-competent haploid germ cells with the birth of healthy progeny (Komeya et al., 2016). Most importantly, when compared to a static agarose gel (AG) organotypic culture system (Sato et al., 2011), the MF system allowed for both an improved tissue survival and a significantly higher number of spermatids and spermatozoa being generated *ex vivo* (Komeya et al., 2016).

Subsequently, the same group later demonstrated that by using a “PDMS cover chip” (PC) to cover and flatten (thus also improving tissue perfusion and oxygenation) the ITT fragment on the classical AG static system, mice spermatogenesis efficiency *in vitro* was also significantly improved (Komeya et al., 2019). However, no comparison with the MF system was done.

The impact of such PDMS-based systems on the IVM of ITT from larger animal species (including humans) remains unknown to date. As marked differences exist between rodent and human spermatogenesis in terms of regulation (e.g., CXCL12-CXCR4 expression, CSF1R localization, Hedgehog/NOTCH signaling) (Guo et al., 2018) and efficiency (Fayomi and Orwig, 2018) it is important to determine whether results obtained in mice can be translated to other species.

In this study we therefore aimed to compare four different organotypic tissue culture systems (3 static and 1 dynamic) using fresh ITT fragments from the domestic pig (a species that has a very close reproductive tract anatomy and physiology to that of humans, with a poorly efficient spermatogenesis) (Swindle and Smith, 1998; Almeida et al., 2006; Groenen et al., 2012), in order to help defining to which extent the type of culture system influences the maturation of large mammalian ITT *in vitro*.

## 2 Materials and Methods

### 2.1 Tissue Culture Systems

Four culture systems were compared in this study as depicted in Table 1.

**TABLE 1:** Summary of culture systems used in the experiments.

Static			Dynamic
Porous PTFE Membrane (0.4 μm Pores)	Agarose gel pillars		Microfluidic PDMS based devices
	Without a PDMS component	With a PDMS component	
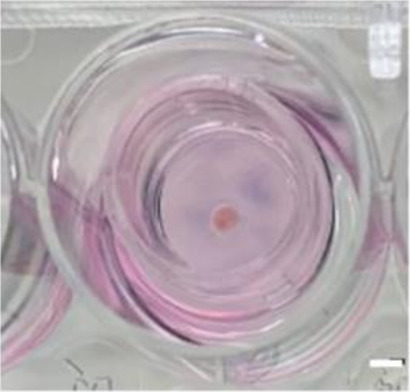	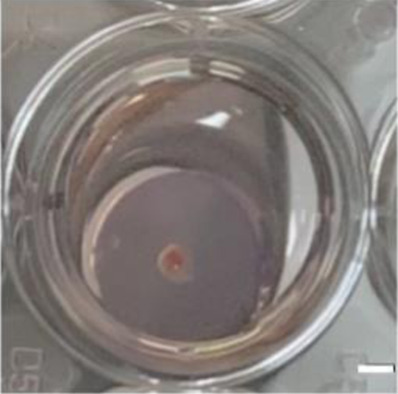	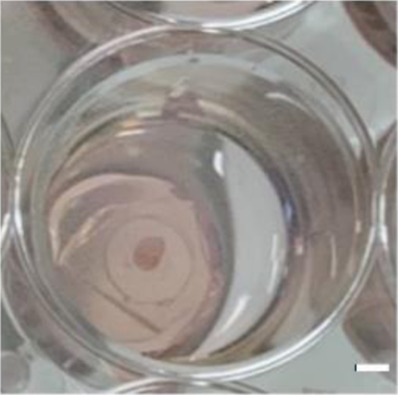	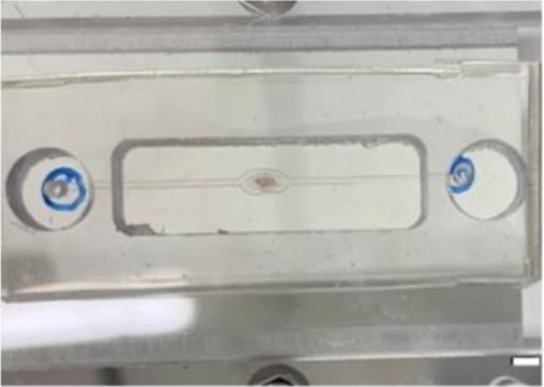
Culture insert PTFE (OT)	Agarose (AG)	Agarose + PDMS cover chip (AGPC)	Microfluidic (MF)

PDMS, Polydimethylsiloxane; PTFE, Polytetrafluoroethylene, Scale bars = 2 mm.

#### 2.1.1 Culture Inserts (OT)

**FIGURE 1:**
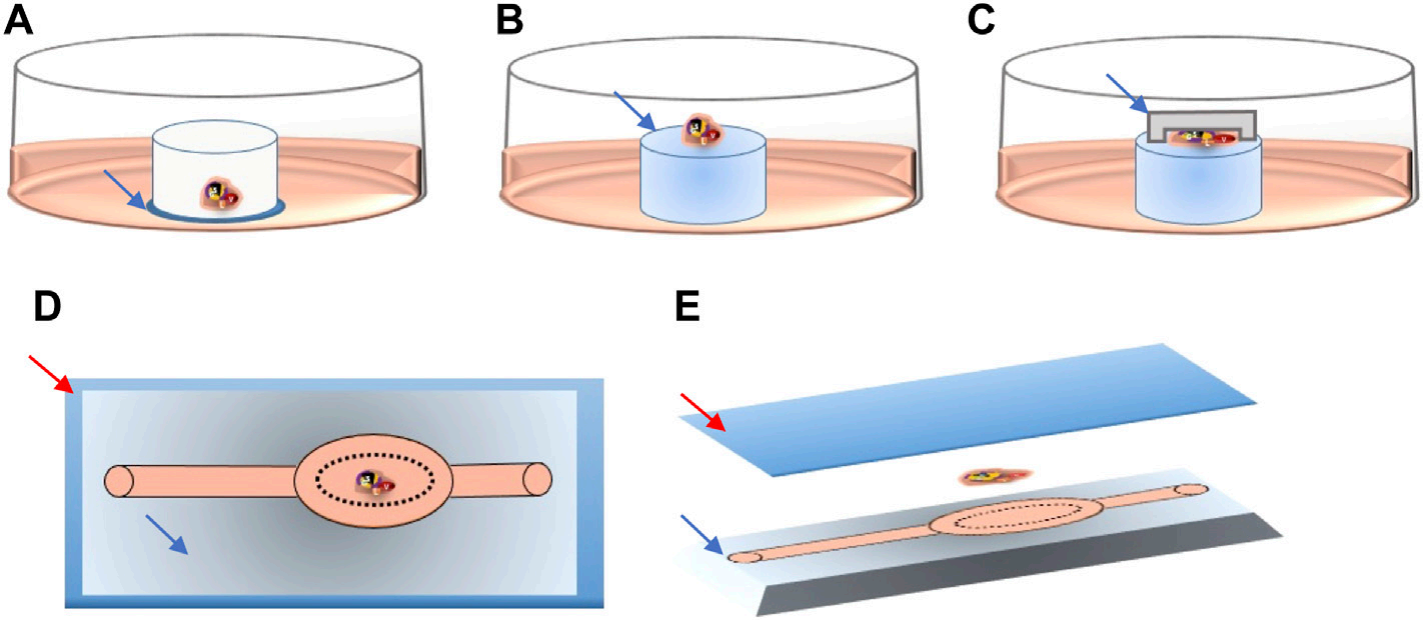
Schematic detail of tissue location in the different culture systems. Culture medium is shown in pink in all systems. **(A)** Blue arrow pointing to the thin PTFE membrane, directly contacting the culture medium. **(B)** Blue arrow pointing to the agarose gel pillar. **(C)** Blue arrow pointing to PDMS cover chip deposited on top of the tissue fragment. **(D)** Upper view of the PDMS chip, red arrow pointing to the glass slide, blue arrow to the patterned PDMS chip. **(E)** 3D lateral view of the PDMS chip showing the glass slide (red arrow), tissue fragment and patterned PDMS chip (blue arrow).

The culture inserts (PICM01250, Merck) used were identical to those previously used by our group to successfully generate haploid human germ cells *in vitro* (de Michele et al., 2018). This system (OT) served therefore as control for all other culture systems. Tissue inserted in this system are in immediate contact with the culture medium (deposited on the thin PTFE membrane) (Figure 1A).

#### 2.1.2 Agarose Gel Pillars (AG)

To make the agarose gel pillars (1.5% w/v), agarose powder (A6013, Sigma Aldrich) was dissolved in distilled water and autoclaved for 30 min. A 5 mm thick layer of agarose gel was formed after pouring and cooling of 30 ml of the agarose liquid solution into a 9.4 cm glass Petri dish. The gel was then perforated using a 10 mm diameter punch biopsy (69036-100, Harris Uni-core) into multiple cylinder-shaped pillars. Pillars were then placed in the wells of 24-well culture plates (CLS3527, Corning Star). They were entirely covered with culture medium and left in the incubator overnight. The next day, at the start of the experiments, the old medium was replaced by fresh medium. Each agarose pillar was then loaded with one testicular tissue fragment. Tissue inserted in this system were not in direct contact with the culture medium but with the agarose surface (Figure 1B).

#### 2.1.3 Agarose Gel Pillars With PDMS Cover Chip (AGPC)

For the AGPC culture system, the PC chip (with a ≃ 170 μm deep chamber) was added on top of the tissue fragments, thus evenly spreading the tissue deposited on the same agarose pillars described above (Figure 1C).

#### 2.1.4 Microfluidic System (MF)

For the MF culture system, tissue fragments were deposited inside the tissue chamber (Figure 1D) of the plasma pre-treated MF chips (SB plasma treater, BlackHoleLab, France). The patterned PDMS chips were then bonded to a microscope glass (VWR, Belgium) and connected to the medium inlet tube (374080, Thermofisher) and syringe-pump outlet (Aladdin NE-1200-EM, WPI, UK) using 1.0/2.0 mm PVC tubes (10192251, FischerSci) (Figures 1D,E).

### 2.2 Design and Fabrication of the PDMS Components for the AGPC and MF Systems

The MF devices and PC chips were fabricated using the photolithography and soft lithography techniques. Protocols used for fabrication were modified from those described by the group of Ogawa (Komeya et al., 2016; Komeya et al., 2019) to achieve a small device that could fit on a standard microscope slide (75 × 25 mm), with only one inlet, and an elliptic-shaped chamber that is perfused from all sides (Supplementary Figure 1).

Briefly, the PDMS prepolymer and curing reagent (Sylgard 184, Dow) were mixed at a 10:1 weight ratio. Then the mixture (10 g for PC and 30 g for MF) was degassed and poured over the specific mold masters (Supplementary Figure 1B, F), which were then placed in an oven at 80 °C for 45 min for curing. After cooling, the solidified PDMS was peeled off from the master mold and was cut using a surgical blade into individual chips. PC and MF chips were about 1 and 3 mm thick, respectively.

Mold masters used for soft lithography were produced as previously reported using conventional photolithography techniques (McDonald et al., 2000). Briefly, the material of the master mold, a negative photoresist (SU-8, 2100; MicroChem Co.) was poured on a 4-inch wafer and spincoated over it to evenly achieve the target thickness of 170 μm over the wafer. After prebaking, the coated wafer was exposed to ultraviolet light (at 365 nm) that was administered through the different photomasks, and then postbaked at 100°C. The baked mold masters were immersed in an SU8-Developer (Y020100, MicroChem) in an ultrasonic bath environment for 20 min and were then rinsed in isopropanol. Wafers were finally silanized prior to use.

The photomasks were designed with CAD software (AutoCAD 2019, academic free license; Autodesk Inc., San Rafael, California) and fabricated with a laser lithography system (JD photomask, United Kingdom).

### 2.3 Animal Tissue and Culture Method

Piglet testes (approx. 5–9 days old) were recovered as a byproduct of castration from a local Belgian swine farm. Briefly, testes (n = 3) were decapsulated, and cut into small fragments (0.5–1 mm^3^) and cultured in the different culture systems at 34°C in 5% CO_2_. Static culture systems were all placed in 24-well plates.

The amount of medium added was 300 µl for the OT, AG, and AGPC groups with change of medium every 4–5 days. For the MF group, medium was drawn through the chip outlet using the syringe pump at a speed of 0.05 μl/min. Fresh culture medium was added to the inlet reservoir in the MF group every 4–5 days. All PDMS and PVC components used for the culture experiments were initially sterilized in an autoclave for 20 min.

The culture medium used in all experiments was Dulbecco’s Modified Eagle Medium: Nutrient Mixture F-12 (DMEM/F-12) supplemented with Knockout serum replacement (KSR) 10%, FSH (5 IU/L), and antibiotics (Gentamicin 10 μg/ml and Ceftazidime 3 mg/L) as previously described by our group (de Michele et al., 2018).

### 2.4 Live Imaging of the Tissue in Culture

Tissues in culture were observed every 5 days with an inverted microscope (Oxion Inverso, EUROMEX, Netherlands) and serial pictures were taken to evaluate the growth of tissue fragments. Images were then processed using the ImageJ software (Schneider et al., 2012) to determine the tissue surface and its 2D evolution through the culture period.

### 2.5 Retrieval of Cultured Tissue Fragments and Supernatants

Cultured ITT fragments from three piglets were harvested for analyses on days 5, 10, 20, and 30 and then fixed in paraformaldehyde 4% (VWR, Belgium) overnight before being embedded in paraffin. Five-micrometer-thick sections on Superfrost^®^ Plus slides (VWR, Belgium) were used for histology, immunohistochemistry (IHC) and immunofluorescence (IF). Supernatants were retrieved every four to 5 days and stored at −80°C.

### 2.6 Histological and Immunohistochemical Analyses

For histological examination, two sections showing the largest cut surface were made for each specimen and stained with Periodic acid-Schiff (PAS) to assess seminiferous tubule (ST) cross-sections (aspect ratio of 1–1.5) for their integrity based on a previously described score (de Michele et al., 2017; de Michele et al., 2018) (Supplementary Figure 2A) and to assess tissue core integrity by measuring centrally degenerated and total tissue areas. Results were reported as the ratio of well-preserved over total ST cross-sections (ST integrity) and as the percentage of centrally degenerated among total tissue area (Tissue core integrity). The evolution of well-preserved ST cross-sections mean diameter was also studied (on 40 ST cross-sections per timing and per system) by measuring their largest diameters (inner to inner measurement from the basal lamina).

Immunohistochemistry (IHC) was performed for the identification of undifferentiated spermatogonia (anti-PGP9.5), Sertoli cells (anti-SOX9), Leydig cells (anti-INSL3), meiotic germ cells (anti-SYCP3), post-meiotic germ cells (anti-CREM) and for the assessment of apoptosis (anti-cleaved-caspase-3) and oxidative stress (Lipid peroxidation biomarker anti-MDA) according to protocols previously described (de Michele et al., 2018; Del Vento et al., 2019; Vermeulen et al., 2019).

Duplex immunofluorescence (IF) imaging was performed to identify proliferating germ cells (anti-Ki67, anti-VASA) and to confirm the identification and location of meiotic (anti-VASA+ and anti-SYCP3+) and post-meiotic (anti-VASA+, anti-CREM+) germ cells. All analyses were performed on two randomly selected sections at least 50 μm apart.

For histology and IHC images, slides were scanned at 400x using a Leica SCN400 slide scanner (Leica Microsystems, Wetzlar, Germany) and for IF using a Zeiss Axio Scan. Z1 (Carl Zeiss, Göttingen, Germany). Quantification was done on entire tissue sections using Qupath software (Bankhead et al., 2017) for all the studied markers. Results for intratubular markers (PGP9.5, SOX9, VASA+/Ki67+, SYCP3 and CREM) were reported as the number of positive cells per ST cross-section (de Michele et al., 2018). Results for Cleaved caspase-3 and INSL3 were reported as the percentage of positive cells among the total cell and total interstitial cell populations, respectively. Results for MDA were reported as a HistoScore (Jensen et al., 2017). The HistoScore was calculated by a semi-quantitative assessment including both the intensity of staining (stained membranes graded as 0, non-staining; 1, weak; 2, median; or 3, strong) and the percentage of positive cells (Supplementary Figure 2B). The following formula was used; HistoScore = ((1 x % weakly stained cells) + (2 x % moderately stained cells) + (3 x % strongly stained cells). The total surface area of tissue sections was considered for analyzes of tissue core integrity, cleaved caspase-3, INSL3 and MDA.

For positive IHC tissue controls, we used neonatal porcine ITT (PGP9.5), tonsil (cleaved caspase-3), mature porcine testicular tissue (for VASA, Ki67, INSL3, SYCP3, and CREM), and human placenta (MDA). For negative controls the primary antibodies were omitted (Supplementary Figure 4).

All primary and secondary antibodies used in these experiments are detailed in Supplementary Table 1.

### 2.7 ELISA Analyses

Reagents of testosterone ELISA kits (ABNOKA2349, Abnova, Taiwan) were prepared following the manufacturer’s instructions. Supernatants were homogenized and centrifuged before analyses. A 50-fold dilution was necessary for supernatants of the OT, AG, and AGPC, groups starting day 10, while a five-fold dilution was applied to supernatants of day 5 samples from all groups and to the MF supernatants at all timepoints. Absorbance was read at 450 nm by the iMark™ microplate absorbance reader (1681135, Bio-Rad).

### 2.8 Statistical Analyses

Statistical analyses were performed with the GraphPad Prism 9 software (GraphPad Software, La Jolla, CA, United States). Data are presented as mean ± SD. A mixed-effects analysis followed by a Dunnett’s test for multiple comparison (*n* = 3 for histology, immunohistochemistry, and ELISA measures) were performed on normally distributed data (with or without a log-transformation) to analyze the effect of culture time and culture system on the different dependent variables. Random effects (within subjects’ variability) for the different piglets were also analyzed within the model and only reported when significant. OT system and day 0 results were used as control. Pearson’s *r* was used for correlation analyses. Statistically significant results were reported as ∗ (*p* ≤ 0.05), ∗∗ (*p* ≤ 0.01), ∗∗∗(*p* ≤ 0.001) and ∗∗∗∗ (*p* ≤ 0.0001) on graphs.

An additional post-hoc multiple comparison (Tukey’s HSD) analysis comparing all systems among each other (and not to control alone) was also performed.

## 3 Results

### 3.1 Evolution of Tissue Size, Seminiferous Tubule Diameter and Integrity, and Tissue Core Integrity in Culture.

**FIGURE 2:**
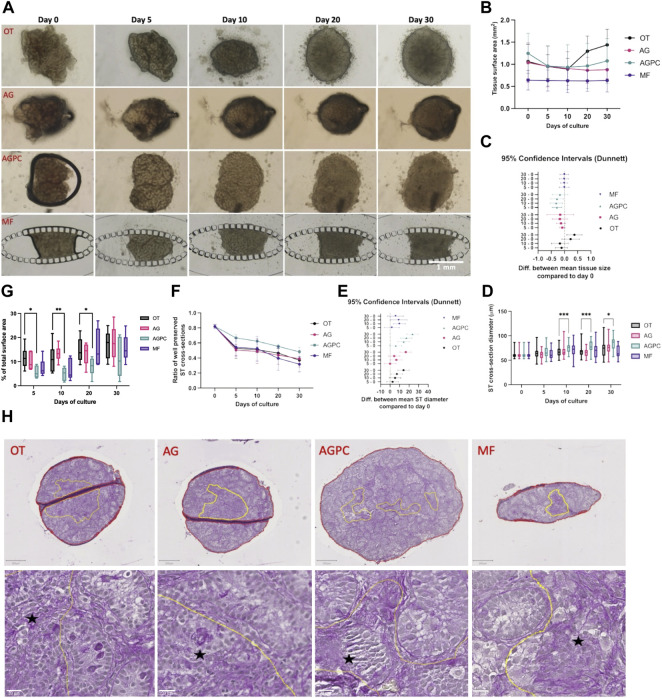
Evolution of tissue size change, ST cross-section diameter and integrity, and tissue core degeneration over the culture period. **(A)** Photographic images of tissue fragments in the different systems from day 0 to day 30. **(B)** Evolution of mean tissue size over time in all systems. **(C)** Difference in mean tissue size between day 30, 20, 10 and day 0. **(D)** Evolution of mean ST cross-sections diameter in the different systems over the culture period. **(E)** Difference in mean ST cross-section diameter between day 30, 20, 10 and day 0. **(F)** Evolution of mean ST cross-sections integrity in the different systems over the culture period. **(G)** Change in the percentage of the central degenerative area (among total tissue area) in the different systems over the culture period. **(H)** Assessement of the integrity of the tissue core on PAS stained slides at day 30. Yellow lines: zone(s) with poor core integrity. Red line(s): total tissue surface area. Black star: areas with altered tissue architecture. OT, culture insert PTFE; AG, Agarose; AGPC, Agarose + PDMS cover, MF, Microfluidic.

The evolution of tissue size over the culture period in the different systems is shown in (Figures 2A,B). Both culture time (*p* = 0.000) and the type of system (*p* = 0.01) were shown to have a significant impact on the results.

By day 30, fragments in the OT (1.3 ± 0.12 mm^2^) system had increased in size (*p* = 0.01) while those in the AG (0.86 ± 0.21 mm^2^), AGPC (1.07 ± 0.5 mm^2^) and MF (0.63 ± 0.26 mm^2^) did not significantly change when compared to day 0 (OT: 1.06 ± 0.39 mm^2^, AG: 1.16 ± 0.24 mm^2^, AGPC: 1.24 ± 0.45, MF: 0.64 ± 0.22 mm^2^). The mean changes in fragments’ area between the different timepoints and the beginning of the culture (day 0) are shown in Figure 2C.

The mean diameter of ST cross-sections statistically increased between day 0 (61.2 ± 6.5 μm) and the end of the culture in all systems (*p* < 0.000) (at day 30, OT: 75.3 ± 18.1 μm, AG: 77.7 ± 15.7 μm and AGPC: 84.9 ± 13.6 μm) except for MF (66.9 ± 12.9 μm) where the increase was only significant till day 20 (71 ± 13 μm, *p* = 0.04). The tissue fragments cultured in the AGPC system had a significantly greater mean ST diameter at days 10 (*p* < 0.000), 20 (*p* < 0.000) and 30 (*p* = 0.02) when compared to control (Figure 2D). The mean changes in ST diameters between the different timepoints and the beginning of the culture (day 0) are shown in Figure 2E.

The analysis for ST integrity was performed on a total of 931 ST cross-sections (OT: 245, AG: 254, AGPC: 228, MF: 204). Culture time (*p* < 0.000) and type of system (*p* = 0.04) were shown to have a significant impact on the results.

There was a significant decrease over time in the ratio of well-preserved ST-cross sections in all systems. When compared to day 0 (0.81 ± 0.03), at day 30 the ratio of well-preserved STs was: 0.37 ± 0.01 (OT, *p* < 0.000), 0.39 ± 0.11 (AG, *p* < 0.000), 0.48 ± 0.06 (AGPC, *p* = 0.000) and 0.31 ± 0.1 (MF, *p* < 0.000). There were no significant differences between any of the systems at any timepoint (Figure 2F). No correlation was found between the mean evolution of tissue fragments’ size and ST integrity in any of the studied systems (OT: r = 0.23, *p* = 0.41, AG: r = 0.48, *p* = 0.41, AGPC: r = 0.56, *p* = 0.32, MF: r = 0.77, *p* = 0.12).

We found a statistically significant effect of time (*p* < 0.000) and type of culture system (*p* = 0.001) on the percentage of tissue core degeneration (Figures 2G,H). Central tissue degeneration was evidenced starting day 5 and increased in all systems reaching mean peak values at day 30 (OT: 17.1 ± 5.79%, AG: 17.75 ± 7.02%, AGPC: 11.41 ± 8.23%, MF: 15.65 ± 5.53%). Tissue fragments cultured in the AGPC system had a significantly smaller percentage of tissue area with core degeneration at days 5 (*p* = 0.03), 10 (*p* = 0.008) and 20 (*p* = 0.03) when compared to control (Figure 2G). The total tissue surface area analyzed was 47.7 mm^2^ (OT: 14.1, AG: 11.9, AGPC: 13.4, MF: 8.2 mm^2^).

Based on the multiple comparison using Tukey’s test among all four systems, tissue fragments cultured with the AGPC system also showed a larger mean ST diameter, when compared to AG (at day 10, *p* = 0.01 and 20, *p* < 0.000) and when compared to MF (at day 30, *p* < 0.000), and an improved tissue core integrity when compared to AG (at day 10, *p* < 0.000) (Supplementary Figure 5).

### 3.2 Undifferentiated Spermatogonia and Sertoli Cell Numbers

**FIGURE 3:**
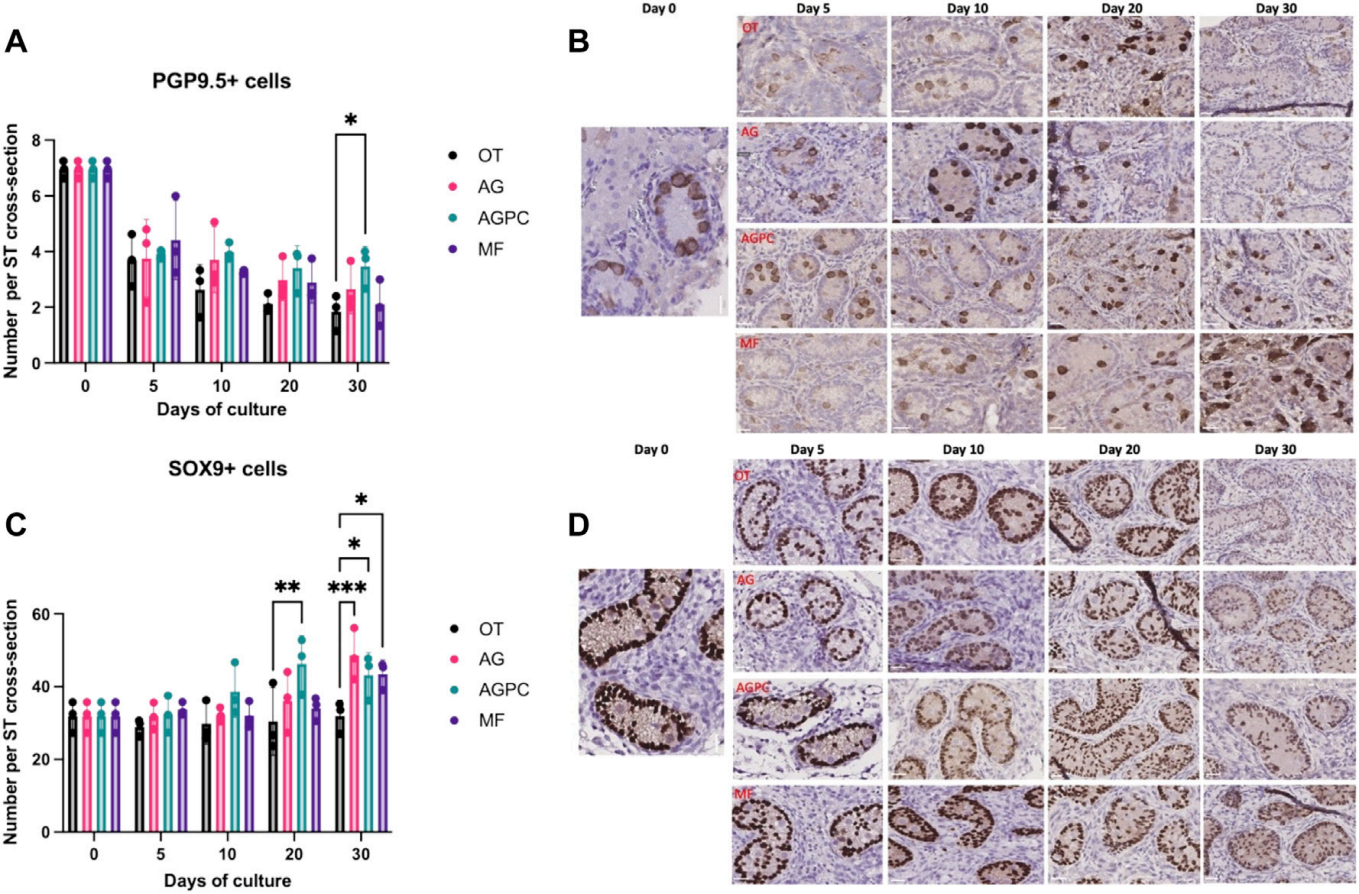
Evolution of undifferentiated spermatogonia and Sertoli cell numbers per ST cross-section over the culture period. **(A)**: Evolution of the number of PGP9.5 positive cells per ST cross-section over the culture period. **(B)**: PGP9.5 staining on immunohistochemistry. **(C)**: Evolution of the number of SOX9 positive cells per ST cross-section over the culture period. **(D)**: SOX9 staining on immunohistochemistry. OT, culture insert PTFE; AG, Agarose; AGPC, Agarose + PDMS cover; MF, Microfluidic. Images shown at ×400 magnification. Scale bars = 20 μm.

The analysis for PGP9.5 cells was performed on a total of 1408 ST cross-sections (OT: 354, AG: 388, AGPC: 398, MF: 268). We found a statistically significant effect on the average number of undifferentiated spermatogonia (PGP9.5+) per ST cross-section of both the culture time (*p* < 0.000) and the culture system (*p* = 0.02) (Figures 3A,B).

Compared to day 0 (6.94 ± 0.31), there was a statistical decrease in the number of PGP9.5 + cells at all time points and in all systems (*p* < 0.000). However, at day 30 (OT: 1.84 ± 0.64, AG: 2.65 ± 0.88, AGPC: 3.72 ± 0.70, MF: 2.11 ± 0.83), the number of PGP9.5 + cells/ST cross-section was found to be higher in the AGPC system in comparison to OT (*p* = 0.01) (Figure 3A).

We found a statistical effect on the average number of Sertoli cells (SOX9+) per ST cross-section (analysis was performed on a total of 882 ST cross-sections, OT: 254, AG: 204, AGPC: 234, MF:190) of both the culture time (*p* < 0.000) and the culture system (*p* = 0.002) (Figures 3A,B).

Between days 0 (31.82 ± 4.25) and 30, the number of Sertoli cells (SOX9+) significantly increased in AG (48.64 ± 7.11, *p* = 0.000), AGPC (43.1 ± 6.14, *p* = 0.001) and MF (43.44 ± 3.86, *p* = 0.001) systems but not in OT (31.93 ± 4.35). At day 30, there were significantly more SOX9+ cells/ST cross-section in the AG (*p* = 0.001), AGPC (*p* = 0.03) and MF (*p* = 0.02) systems in comparison to control (OT) (Figures 3C,D).

Also, the multiple comparison analysis using Tukey’s test showed that the number of SOX9+ cells/ST was significantly higher in the AGPC group in comparison to MF (day 20, *p* = 0.03) (Supplementary Figure 5).

### 3.3 Germ Cell Proliferation and Differentiation

**FIGURE 4:**
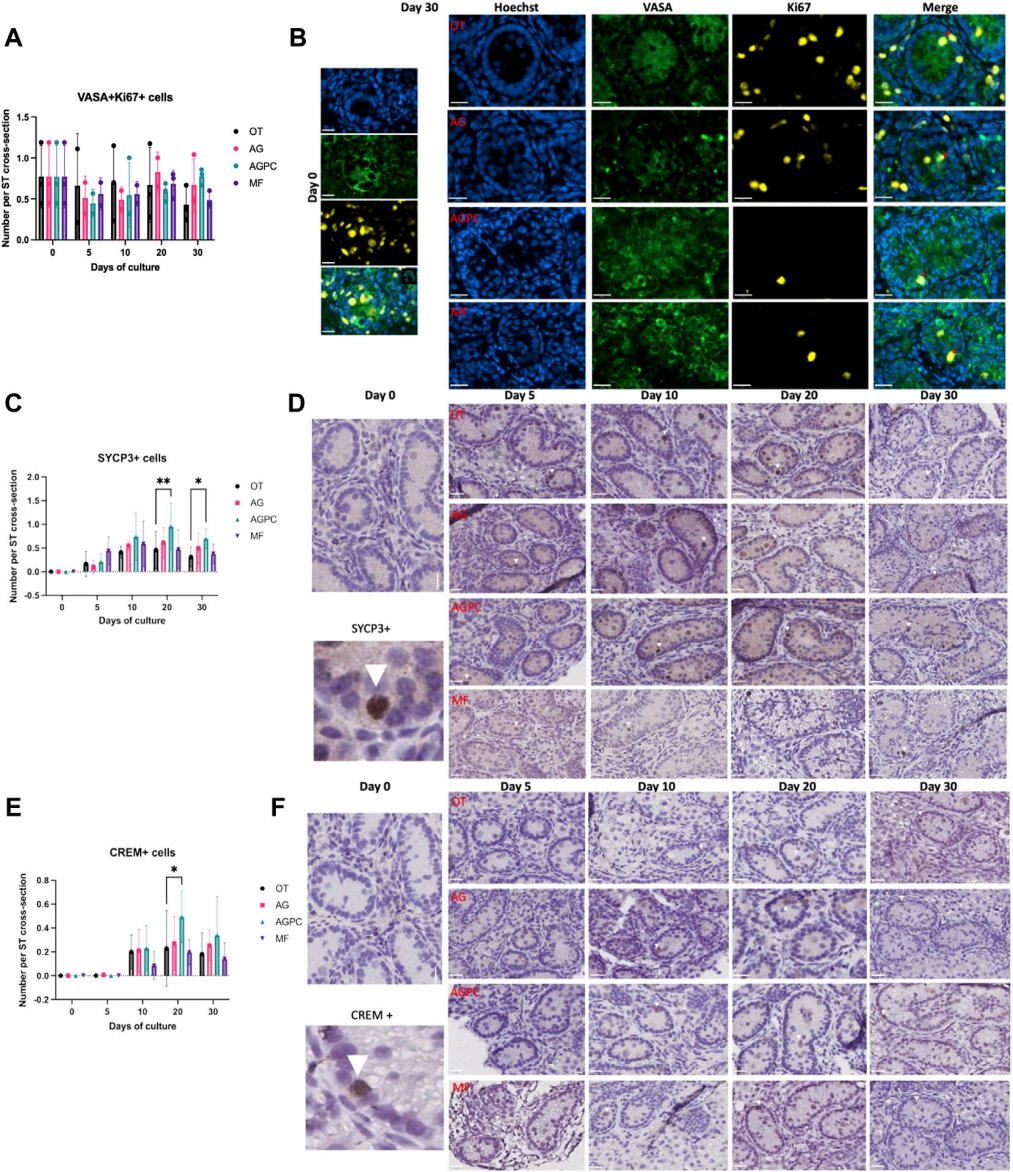
Evolution of germ cell proliferation and differentiation over the culture period. **(A)** Evolution of the number of proliferating germ cells (VASA + Ki67+) cells per ST cross-section over the culture period. **(B)** Immunofluorescence duplex staining for VASA/Ki67, red arrows indicate proliferating germ cells. **(C)** Evolution of the number of meiotic (SYCP3+) cells per ST cross-section over the culture period. **(D)** SYCP3 staining on immunohistochemistry, white arrowheads show SYCP3+ staining. **(E)** Evolution of the number of post-meiotic (CREM+) cells per ST cross-section over the culture period, **(F)** CREM staining on immunohistochemistry, white arrowheads show CREM + staining. OT, culture insert PTFE; AG, Agarose; AGPC, Agarose + PDMS cover; MF, Microfluidic. Images shown at ×400 magnification. Scale bars = 20 μm.

Both the culture time and the culture system did not have a significant impact on germ cell proliferation (VASA + Ki67 + cells). Changes in the mean number of VASA + Ki67 + per ST cross-section were insignificant between day 0 (0.77 ± 0.38) and day 30 (OT: 0.43 ± 0.21, AG: 0.66 ± 0.32, AGPC: 0.77 ± 0.1, MF: 0.48 ± 0.1) (Figures 4A,B). The analysis for VASA + Ki67 + cells was performed on a total of 1107 ST cross-sections (OT: 307, AG: 285, AGPC: 335, MF: 180).

Spermatocytes (SYCP3+) were absent at day 0 in ITT of all piglets (Figures 4C,D). Culture time (*p* < 0.000) and the type of system (*p* = 0.006) were shown to have a significant impact on their generation.

Spermatocytes were observed as early as day 5, and their numbers increased significantly starting day 10 in all four systems (OT: 0.41 ± 0.26, *p* = 0.04, AG: 0.55 ± 0.1, *p* = 0.003, AGPC: 0.74 ± 0.49, *p* < 0.000, MF: 0.58 ± 0.4, *p* = 0.04) (Figure 4C). At days 20 and 30, the number of SYCP3+ cells/ST cross-section in the AGPC system (d20; 0.95 ± 0.49, d30; 0.69 ± 0.21) was statistically higher than in control (d20; 0.46 ± 0.39, d30; 0.31 ± 0.21) (d20; *p* = 0.007, d30; *p* = 0.04) (Figures 4C,D). The analysis for SYCP3 was performed on a on a total of 1524 ST cross-sections (OT: 416, AG: 362, AGPC: 457, MF: 289).

Post-meiotic germ cells (CREM+) were not observed on day 0 in any of the piglets (Figures 4E,F). They started to develop at day 10 in the different systems (except for a few positive cells observed starting day 5 in the AGPC group). At day 30, the average number of CREM + cells per ST cross-section was: OT (0.18 ± 0.17), AG (0.25 ± 0.13), AGPC (0.34 ± 0.32) and MF (0.14 ± 0.13).

Although there was a higher number of CREM + cells/ST cross-section in the AGPC group at day 20 in comparison to control (*p* = 0.03), there were no differences observed between the four systems at day 30. Only culture time (*p* < 0.000) and not the type of culture system (=0.06) had an impact on their overall development.

The analysis for CREM was performed on a total of 1293 ST cross-sections (OT: 405, AG: 295, AGPC: 318, MF: 275).

The double IF staining preformed for SYCP3 and CREM with VASA confirmed the expression of both protein markers in differentiating germ cells (Supplementary Figure 3).

### 3.4 Oxidative Stress and Apoptotic Cell Death

**FIGURE 5:**
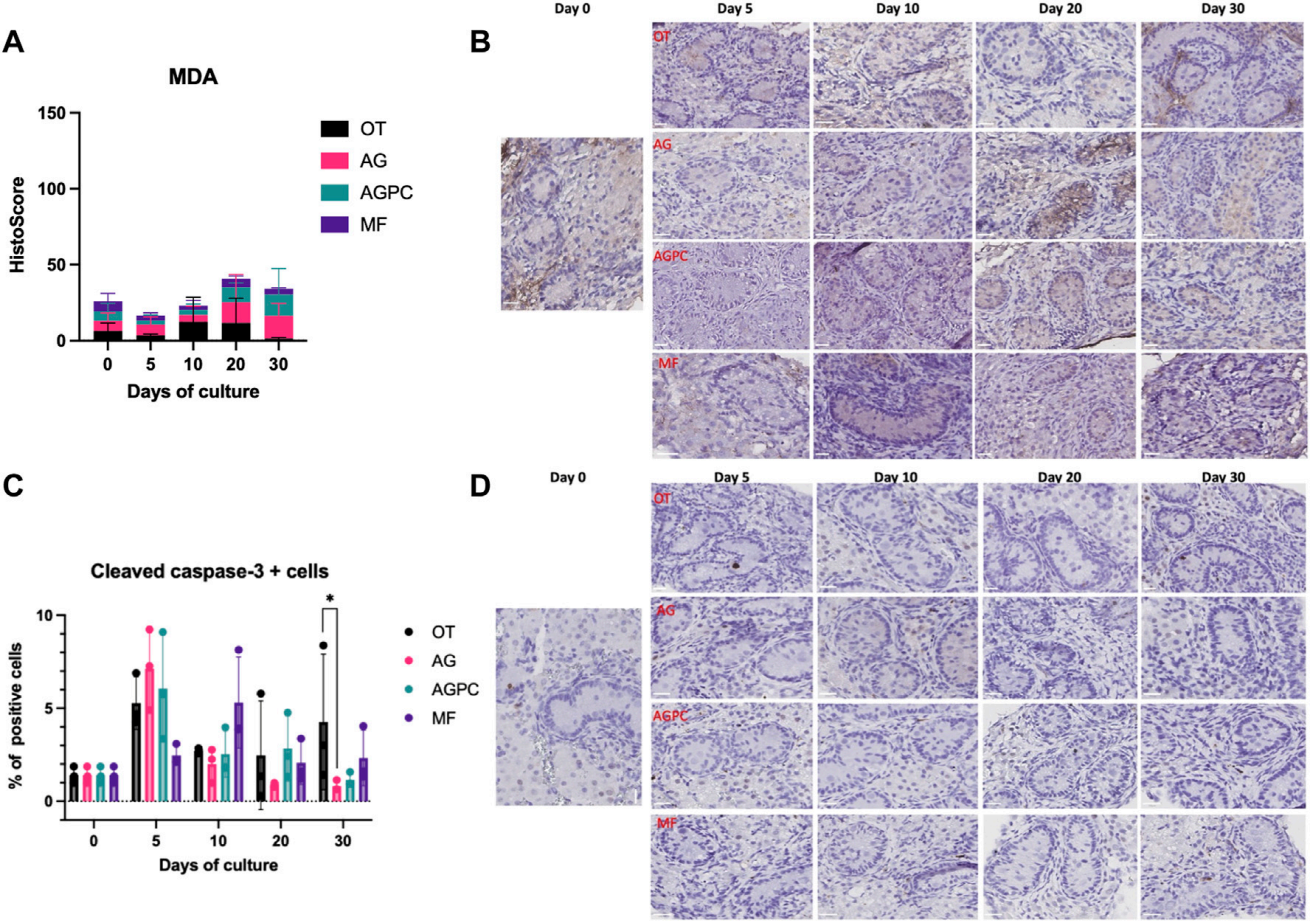
Evolution of apoptosis and oxidative stress markers over the culture period. **(A)** Evolution of the MDA staining (HistoScore) levels over the culture period. **(B)** MDA staining on immunohistochemistry. **(C)** Evolution of the percentage of cleaved caspase-3 positive cells over the culture period. **(D)** Cleaved caspase-3 immunohistochemistry. OT, culture insert PTFE; AG, Agarose; AGPC, Agarose + PDMS cover; MF, Microfluidic. Images shown at ×400 magnification, Scale bars = 20 μm.

Based on the HistoScore evaluation, we found that levels of MDA (Figures 5A,B) did not vary significantly neither over the culture period nor among the different systems. There was no difference between the HistoScore value for MDA level at day 0 (6.45 ± 5.28) and at day 30 for the different systems (OT: 1.41 ± 0.72, AG: 14.9 ± 8.19, AGPC: 13.95 ± 17.21, MF: 3.96 ± 0.63). The total tissue surface area analyzed was 39.95 mm^2^ for MDA assessment (OT: 10.84, AG: 9.47, AGPC: 10.82, MF: 8.82 mm^2^).

The percentage of cleaved caspase-3 positive cells (Figures 5C,D) did not vary with the type of culture system but was significantly influenced by the culture time (*p* < 0.000). Compared to day 0 (1.4 ± 0.44%), a significant peak in apoptosis was noted at day 5 in the static systems (OT: 5.28 ± 1.41%, *p* = 0.02; AG: 7.13 ± 2.16%, *p* < 0.000, and AGPC: 6.07 ± 6.87%, *p* = 0.005) and at day 10 in the dynamic system (MF: 5.31 ± 2.45%, *p* = 0.01) (Figure 5D). At day 30, the percentage of cleaved caspase-3 positive cells was statistically lower in the AG system in comparison to control (*p* = 0.006). The total tissue surface area analyzed was 41.1 mm^2^ for cleaved caspase-3 evaluation (OT: 9.73, AG: 11.23, AGPC: 12.41, MF: 7.74 mm^2^).

### 3.5 Leydig Cell Numbers and Testosterone Secretion

**FIGURE 6:**
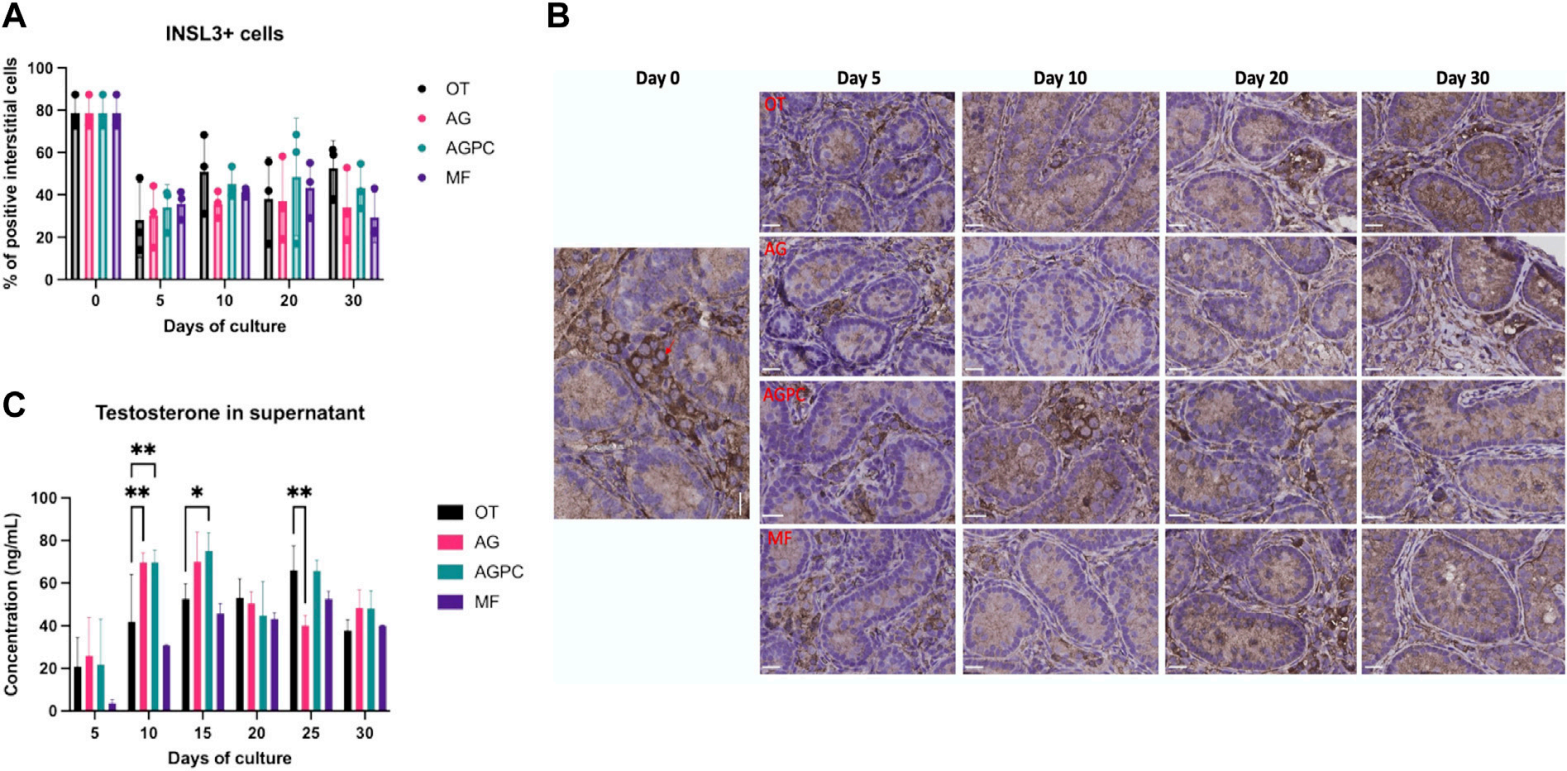
Evolution of Leydig cell numbers and function over the culture period. **(A)** Evolution of the percentage of INSL3+ interstitial cells over the culture period. **(B)** INSL3 staining on immunohistochemistry. Red arrow shows a positive cell. **(C)** Evolution of testosterone secretion over the culture period. OT, culture insert PTFE; AG, Agarose; AGPC, Agarose + PDMS cover; MF, Microfluidic. Images shown at ×400 magnification, Scale bars = 20 μm.

We found a statistically significant effect of time (*p* < 0.000) on the number of Leydig cells (INSL3+) though the effect of the type of culture system was not significant (Figures 6A,B).

At day 0, INSL3+ cells represented an average of 78.66 ± 7.67% of all interstitial cells. By culture day 5, the percentage of INSL3+ stained cells in the interstitium significantly dropped in all systems (OT: 28.11 ± 17.7, AG: 30.38 ± 14.6, AGPC: 34.2 ± 10.6, MF: 35.9 ± 6.83%) and remained unchanged till day 30 (Figure 6A). The total tissue surface area was 45.11 mm^2^ for INSL3 analyses (OT: 13.9, AG: 10.51, AGPC: 12.8, MF: 7.9 mm^2^).

We found a significant effect of both culture time (*p* < 0.000) and the culture system (*p* = 0.01) on the testosterone concentrations in culture supernatants. Testosterone secretion increased in all systems (Figure 6C). When compared to day 5, testosterone levels at the end of the culture period (day 30) were significantly higher in AG: 48.4.62 ± 8.5, *p* = 0.01, AGPC: 48.2 ± 8.4, *p* = 0.006, and MF: 40.1 ± 0.3 ng/ml, *p* < 0.000 but not in OT: 37.9 ± 5 ng/ml. At day 30, there were no significant differences in testosterone concentrations between any of the culture systems (Figure 6C). Peaks for testosterone levels were observed at days 15 (for AG: 70.1 ± 13.8 and AGPC: 75.2 ± 8.4 ng/ml) and 25 (for OT: 66 ± 11.4 and MF: 52.7 ± 3.5 ng/ml).

When all four systems were compared among each other (Tukey’s test), testosterone levels were found statistically higher in the AGPC group compared to MF at days 10 (*p* < 0.000) and 15 (*p* = 0.01) and compared to AG on day 25 (*p* = 0.007) (Supplementary Figure 5).

No correlation was found between the % of INSL3+ cells and testosterone in any of the studied systems (OT: r = 0.03, *p* = 0.93, AG: r = -0.13, *p* = 0.67, AGPC: r = 0.16, *p* = 0.62, MF: r = 0.06, *p* = 0.87).

## 4 Discussion and Conclusion

In 2018 we demonstrated for the first time that haploid human germ cells (round spermatids) can be generated *in vitro* (after 16 days) when ITT fragments were cultured on PTFE culture inserts (OT) using an enriched culture medium containing KSR 10% and FSH 5 IU/L (de Michele et al., 2018). This was an important first step for the future clinical translation of IVM as a fertility restoration strategy.

As these haploid cells were generated in low numbers, and as murine spermatogenesis *ex vivo* was enhanced when the type of physical culture approach was modified (Komeya et al., 2016; Komeya et al., 2019), it became clear that further studies exploring the impact of the type of culture system, regardless of the culture media components, on the spermatogenic process *ex vivo* were needed.

Due to the scarcity of human ITT available for research, in this work, we chose the porcine model, a large mammal closely related to humans (Swindle and Smith, 1998; Meurens et al., 2012) to study the impact of four different culture systems (OT, AG, AGPC and MF) on the outcome of IVM of ITT in terms of tissue growth, survival and function, including germ cell differentiation. This makes this study the first to report the application of PDMS-based microfluidic (MF) and static (AGPC) organotypic tissue culture systems to ITT of a species that is different from rodents (i.e., mice, rats).

The serial imaging performed in our study was very informative in following the evolution of ITT fragments over time. However, we did not observe a positive relationship between the tissue fragment’s growth and overall ST integrity. Indeed, the increase in tissue surface area noted for the OT group did not correlate with an improved outcome in terms of ST integrity. Such enlargement could be due either to cytotoxic cell edema (Liang et al., 2007), fibrosis-related extracellular matrix remodeling (Bigaeva et al., 2019) or to the fact that cells attach and spread better to the PTFE membrane in the OT system in contrast to agarose and PDMS that are both characterized by low to no cell adhesion (Chuah et al., 2015; Cambria et al., 2020).

The overall presence of tissue core degeneration, loss of both the ST integrity and undifferentiated spermatogonia numbers, as well as the increase in apoptosis over the culture period were disappointing, although not surprising. Such phenomena are still a hurdle in *in vitro* experiments across all species, including mice, rats, non-human primates, and humans (Reda et al., 2016; de Michele et al., 2017; Medrano et al., 2018; Portela et al., 2019a; Portela et al., 2019b; Abe et al., 2020; Heckmann et al., 2020; Kurek et al., 2021; Sharma et al., 2022).

A damaged testicular niche environment (demonstrated by the loss of basal membrane protein LAMA1 *in vitro*) (Kurek et al., 2021) and the massive inflammatory reaction described during organotypic culture (Abe et al., 2020) could both participate to the worsening of the tissue evolution in culture. These factors will need to be considered in future organotypic culture experiments.

However, albeit the decrease in the ratio of well-preserved STs and in undifferentiated spermatogonia numbers, some STs were able to grow (based on the observed increase in diameters over the culture period) and to support undifferentiated spermatogonia proliferation and differentiation. Similarly, Sertoli cell (SOX9+) numbers per ST cross-section also rose and testosterone secretion increased reaching levels up to 100 times higher than normal physiological serum levels (Colenbrander et al., 1978; Bonneau et al., 1987). All these changes are the hallmark of the pubertal transition *in vivo* in pigs (Franca et al., 2000; Koskenniemi et al., 2017) and confirm the successful maturation of the neonatal ITT *in vitro*.

Similar to other studies (de Michele et al., 2018; Heckmann et al., 2020; Sharma et al., 2022), germ cell differentiation progressed as expected (first SYCP3 and then CREM) and was accelerated *in vitro*. While both spermatocytes and presumptive spermatids were observed in all systems, their numbers per ST cross-section were higher in the AGPC group. Also, CREM + cells started appearing at day 5 in the AGPC system, earlier than observed in OT, AG, and MF. Both the accelerated development of presumptive spermatids and their presence in higher numbers in the AGPC system could be attributed to an improvement in nutrient diffusion linked to the flattening of the tissue (and thus the delivery of crucial factors/hormones to a broader surface of tissue) as evidenced by the smaller percentage of degenerative tissue core area observed. Such observations are similar to what was previously reported by the group of Ogawa (Kojima et al., 2018; Komeya et al., 2019).

The early reduction and subsequent stabilization in INSL3+ LC numbers together with the increase in testosterone secretion over the culture period were an interesting finding.

INSL3 is a peptide that is secreted by both fetal and adult-type LCs and is an accurate reflector of LCs’ differentiation status and of their absolute numbers (Ivell et al., 2013). As fetal LCs are known to peak in numbers shortly after birth and then involute as they are progressively replaced by testosterone-secreting adult-type LCs (immature and then mature LCs as puberty progresses) (Van Straaten and Wensing, 1978; Griswold and Behringer, 2009), we assume that a similar phenomenon might have occurred in our experiments *in vitro*. However, as the INSL3+ cells did not re-increase in numbers (as observed during puberty), it is possible that adult LC proliferation was limited in our experiments. Because LCs play a pivotal role in spermatogenesis, their function, and differentiation status in *ex vivo* ITT culture experiments should be better explored in future studies.

Concerning the lipid peroxidation markers, the reactive aldehyde MDA was already expressed at day 0. This is possibly due to the cold storage and transport that is known to lead to glutathione loss (Vreugdenhil et al., 1991). As values of MDA remained stable over the culture period and were not different between systems, we may hypothesize that the presence of glutathione reductase and other antioxidants within the culture medium used (Price et al., 1998) may have played a role and hidden any potential benefit of the PDMS components that shield the tissue fragments from direct contact with ambient oxygen (as a source of reactive oxygen species responsible of oxidative stress).

Our results with the silicone-based MF and AGPC systems are important and encouraging but were modest compared to achievements in IVM of mice ITT. Interestingly, while analyzing our findings, a report on the application of the AGPC system for IVM of rat ITT (Matsumura et al., 2021) also pointed to a limited efficiency of the system in comparison to the previous experiments in mice. In fact, not only was rat spermatogenesis blocked at the round spermatid stage, but under some oxygen conditions the AGPC system lost its added value (reported in the original studies with mice ITT) in comparison the classical AG approach. One may argue that this could be due to inadequacy of culture media components although in the later study an enriched culture medium was used.

These observations as well as ours, strengthen the fact that culture conditions (e.g., physical conditions i.e., systems, timing, culture medium) for *in vitro* spermatogenesis are not translatable from one species to another. Species-specific culture conditions, notably for humans, should therefore be tailored in the future.

While we consider the results of this work as an added value to the current available literature on IVM of ITT, our comparative study has some limitations that should be taken into consideration. Tissue fragments were heterogeneous in size with smaller sizes in the MF system. This means that smaller fragments could have sustained greater damage during dissection (leading to a poorer outcome in terms of tissue core degeneration with the MF device compared to AGPC) while larger fragments might have suffered from poorer nutrient perfusion.

Culture media perfusion was also different between the static and the MF system and could have interfered with interpretation of some of our results. For example, the testosterone level was low at day 5 in the MF group, most probably not because the LCs were not functional but just because the PVC tubes had to be filled with culture medium prior to the experiment start, leading to a diluted testosterone level in this first recovered supernatant sample (that contains media perfused from day 0 to day 5).

As for germ cell differentiation, while both VASA + SYCP3+ and VASA + CREM + germ cells appeared during culture, their overall numbers were low, and their appearance did not occur in clusters as observed *in vivo*. Reduced efficiency of spermatogenesis *in vitro* is a known phenomenon in mice (Komeya et al., 2019), and an even greater challenge in rats (Reda et al., 2016; Matsumura et al., 2021) or larger mammals (Reda et al., 2016; de Michele et al., 2018; Medrano et al., 2018) and should be the focus of future work in the field of *in vitro* spermatogenesis.

Altogether, we were able to show that the porcine ITT underwent functional maturation, and that spermatogenesis was successfully initiated in tissue fragments (from all piglets) cultured in all four systems *in vitro*. Also, we demonstrated that only the AGPC system outperformed the control (OT), mainly by moderately improving both undifferentiated spermatogonia survival and the efficiency of germ cell differentiation.

It is however important to note that repeat experiments with a higher number of pigs/samples to increase the statistical power might allow to further highlight the differences among all four systems.

We conclude that prior to applying complex organotypic tissue culture systems to the IVM of human ITT, future studies should first focus on improving media components (e.g., growth factors, dosage, timing of administration, etc.) as this seems to be the most important limiting factor for the success of IVM at this stage. It is however not excluded that after an optimal culture medium is established, applying MF and AGPC or other complex culture systems could help in improving the efficiency of spermatogenesis *ex vivo*.

## Data Availability

The raw data supporting the conclusion of this article will be made available by the authors, without undue reservation.

## References

[B1] Abe T. Nishimura H. Sato T. Suzuki H. Ogawa T. Suzuki T. (2020). Transcriptome Analysis Reveals Inadequate Spermatogenesis and Immediate Radical Immune Reactions during Organ Culture *In Vitro* Spermatogenesis. Biochem. Biophysical Res. Commun. 530 (4), 732–738. 10.1016/j.bbrc.2020.06.161 32782148

[B2] Abofoul-Azab M. AbuMadighem A. Lunenfeld E. Kapelushnik J. Shi Q. Pinkas H. (2018). Development of Postmeiotic Cells *In Vitro* from Spermatogonial Cells of Prepubertal Cancer Patients. Stem Cells Dev. 27 (15), 1007–1020. 10.1089/scd.2017.0301 29779447

[B3] Almeida F. F. L. Leal M. C. França L. R. (2006). Testis Morphometry, Duration of Spermatogenesis, and Spermatogenic Efficiency in the Wild Boar (*Sus scrofa* Scrofa)1. Biol. Reprod. 75 (5), 792–799. 10.1095/biolreprod.106.053835 16870941

[B4] Baert Y. Ruetschle I. Cools W. Oehme A. Lorenz A. Marx U. (2020). A Multi-Organ-Chip Co-culture of Liver and Testis Equivalents: a First Step toward a Systemic Male Reprotoxicity Model. Hum. Reprod. 35 (5), 1029–1044. 10.1093/humrep/deaa057 32390056

[B5] Bankhead P. Loughrey M. B. Fernández J. A. Dombrowski Y. McArt D. G. Dunne P. D. (2017). QuPath: Open Source Software for Digital Pathology Image Analysis. Sci. Rep. 7 (1), 16878. 10.1038/s41598-017-17204-5 29203879 PMC5715110

[B6] Bigaeva E. Gore E. Simon E. Zwick M. Oldenburger A. de Jong K. P. (2019). Transcriptomic Characterization of Culture-Associated Changes in Murine and Human Precision-Cut Tissue Slices. Arch. Toxicol. 93 (12), 3549–3583. 10.1007/s00204-019-02611-6 31754732

[B7] Bonneau M. Carrié-Lemoine J. Prunier A. Garnier D. H. Terqui M. (1987). Age-related Changes in Plasma LH and Testosterone Concentration Profiles and Fat 5α-Androstenone Content in the Young Boar. Animal Reproduction Sci. 15 (3), 241–258. 10.1016/0378-4320(87)90046-7

[B8] Cambria E. Brunner S. Heusser S. Fisch P. Hitzl W. Ferguson S. J. (2020). Cell-Laden Agarose-Collagen Composite Hydrogels for Mechanotransduction Studies. Front. Bioeng. Biotechnol. 8, 346. 10.3389/fbioe.2020.00346 32373605 PMC7186378

[B9] Chuah Y. J. Koh Y. T. Lim K. Menon N. V. Wu Y. Kang Y. (2015). Simple Surface Engineering of Polydimethylsiloxane with Polydopamine for Stabilized Mesenchymal Stem Cell Adhesion and Multipotency. Sci. Rep. 5, 18162. 10.1038/srep18162 26647719 PMC4673458

[B10] Colenbrander B. de Jong F. H. Wensing C. J. G. (1978). Changes in Serum Testosterone Concentrations in the Male Pig during Development. Reproduction 53 (2), 377–380. 10.1530/jrf.0.0530377 690987

[B11] de Michele F. Poels J. Vermeulen M. Ambroise J. Gruson D. Guiot Y. (2018). Haploid Germ Cells Generated in Organotypic Culture of Testicular Tissue from Prepubertal Boys. Front. Physiol. 9 (1413), 1413. 10.3389/fphys.2018.01413 30356879 PMC6190924

[B12] de Michele F. Poels J. Weerens L. Petit C. Evrard Z. Ambroise J. (2017). Preserved Seminiferous Tubule Integrity with Spermatogonial Survival and Induction of Sertoli and Leydig Cell Maturation after Long-Term Organotypic Culture of Prepubertal Human Testicular Tissue. Hum. Reprod. 32 (1), 32–45. 10.1093/humrep/dew300 27927847

[B13] Del Vento F. Vermeulen M. Ucakar B. Poels J. des Rieux A. Wyns C. (2019). Significant Benefits of Nanoparticles Containing a Necrosis Inhibitor on Mice Testicular Tissue Autografts Outcomes. Ijms 20 (23), 5833. 10.3390/ijms20235833 PMC6929043 31757040

[B14] Dores C. Rancourt D. Dobrinski I. (2015). Stirred Suspension Bioreactors as a Novel Method to Enrich Germ Cells from Pre-pubertal Pig Testis. Andrology 3 (3), 590–597. 10.1111/andr.12031 25877677 PMC4495971

[B15] Fayomi A. P. Orwig K. E. (2018). Spermatogonial Stem Cells and Spermatogenesis in Mice, Monkeys and Men. Stem Cell Res. 29, 207–214. 10.1016/j.scr.2018.04.009 29730571 PMC6010318

[B16] Fayomi A. P. Peters K. Sukhwani M. Valli-Pulaski H. Shetty G. Meistrich M. L. (2019). Autologous Grafting of Cryopreserved Prepubertal Rhesus Testis Produces Sperm and Offspring. Science 363 (6433), 1314–1319. 10.1126/science.aav2914 30898927 PMC6598202

[B17] França L. R. Silva V. A. Jr. Chiarini-Garcia H. Garcia S. K. Debeljuk L. (2000). Cell Proliferation and Hormonal Changes during Postnatal Development of the Testis in the Pig. Biol. Reprod. 63 (6), 1629–1636. 10.1095/biolreprod63.6.1629 11090429

[B18] Goossens E. Jahnukainen K. Mitchell R. van Pelt A. Pennings G. Rives N. (2020). Fertility Preservation in Boys: Recent Developments and New Insights. Hum. Reprod. Open 2020 (3), hoaa016. 10.1093/hropen/hoaa016 32529047 PMC7275639

[B19] Griswold S. L. Behringer R. R. (2009). Fetal Leydig Cell Origin and Development. Sex. Dev. 3 (1), 1–15. 10.1159/000200077 19339813 PMC4021856

[B20] Groenen M. A. Archibald A. L. Uenishi H. Tuggle C. K. Takeuchi Y. Rothschild M. F. (2012). Analyses of Pig Genomes Provide Insight into Porcine Demography and Evolution. Nature 491 (7424), 393–398. 10.1038/nature11622 23151582 PMC3566564

[B21] Guo J. Grow E. J. Mlcochova H. Maher G. J. Lindskog C. Nie X. (2018). The Adult Human Testis Transcriptional Cell Atlas. Cell Res. 28 (12), 1141–1157. 10.1038/s41422-018-0099-2 30315278 PMC6274646

[B22] Heckmann L. Langenstroth-Röwer D. Wistuba J. Portela J. M. D. van Pelt A. M. M. Redmann K. (2020). The Initial Maturation Status of Marmoset Testicular Tissues Has an Impact on Germ Cell Maintenance and Somatic Cell Response in Tissue Fragment Culture. Mol. Hum. Reprod. 26 (6), 374–388. 10.1093/molehr/gaaa024 32236422

[B23] Hermann B. P. Sukhwani M. Winkler F. Pascarella J. N. Peters K. A. Sheng Y. (2012). Spermatogonial Stem Cell Transplantation into Rhesus Testes Regenerates Spermatogenesis Producing Functional Sperm. Cell Stem Cell 11 (5), 715–726. 10.1016/j.stem.2012.07.017 23122294 PMC3580057

[B24] Ivell R. Wade J. D. Anand-Ivell R. (2013). INSL3 as a Biomarker of Leydig Cell Functionality. Biol. Reproduction 88 (6), 147. 10.1095/biolreprod.113.108969 23595905

[B25] Jensen K. Krusenstjerna-Hafstrøm R. Lohse J. Petersen K. H. Derand H. (2017). A Novel Quantitative Immunohistochemistry Method for Precise Protein Measurements Directly in Formalin-Fixed, Paraffin-Embedded Specimens: Analytical Performance Measuring HER2. Mod. Pathol. 30 (2), 180–193. 10.1038/modpathol.2016.176 27767098

[B26] Kanbar M. de Michele F. Giudice M. G. Desmet L. Poels J. Wyns C. (2021). Long-term Follow-Up of Boys Who Have Undergone a Testicular Biopsy for Fertility Preservation. Hum. Reprod. 36 (1), 26–39. 10.1093/humrep/deaa281 33259629

[B27] Khong Y. M. Zhang J. Zhou S. Cheung C. Doberstein K. Samper V. (2007). Novel Intra-tissue Perfusion System for Culturing Thick Liver Tissue. Tissue Eng. 13 (9), 2345–2356. 10.1089/ten.2007.0040 17708717

[B28] Kojima K. Nakamura H. Komeya M. Yamanaka H. Makino Y. Okada Y. (2018). Neonatal Testis Growth Recreated *In Vitro* by Two‐dimensional Organ Spreading. Biotechnol. Bioeng. 115 (12), 3030–3041. 10.1002/bit.26822 30144353 PMC6283240

[B29] Komeya M. Kimura H. Nakamura H. Yokonishi T. Sato T. Kojima K. (2016). Long-term *Ex Vivo* Maintenance of Testis Tissues Producing Fertile Sperm in a Microfluidic Device. Sci. Rep. 6, 21472. 10.1038/srep21472 26892171 PMC4759809

[B30] Komeya M. Yamanaka H. Sanjo H. Yao M. Nakamura H. Kimura H. (2019). *In Vitro* spermatogenesis in Two‐dimensionally Spread Mouse Testis Tissues. Reprod. Med. Biol. 18 (4), 362–369. 10.1002/rmb2.12291 31607796 PMC6780044

[B31] Koskenniemi J. J. Virtanen H. E. Toppari J. (2017). Testicular Growth and Development in Puberty. Curr. Opin. Endocrinol. Diabetes Obes. 24 (3), 215–224. 10.1097/MED.0000000000000339 28248755

[B32] Kurek M. Åkesson E. Yoshihara M. Oliver E. Cui Y. Becker M. (2021). Spermatogonia Loss Correlates with LAMA 1 Expression in Human Prepubertal Testes Stored for Fertility Preservation. Cells 10 (2), 241. 10.3390/cells10020241 33513766 PMC7911157

[B33] Liang D. Bhatta S. Gerzanich V. Simard J. M. (2007). Cytotoxic Edema: Mechanisms of Pathological Cell Swelling. Foc 22 (5), 1–9. 10.3171/foc.2007.22.5.3 PMC2740913 17613233

[B34] Lovett M. Lee K. Edwards A. Kaplan D. L. (2009). Vascularization Strategies for Tissue Engineering. Tissue Eng. Part B Rev. 15 (3), 353–370. 10.1089/ten.TEB.2009.0085 19496677 PMC2817665

[B35] Matsumura T. Sato T. Abe T. Sanjo H. Katagiri K. Kimura H. (2021). Rat *In Vitro* Spermatogenesis Promoted by Chemical Supplementations and Oxygen-Tension Control. Sci. Rep. 11 (1), 3458. 10.1038/s41598-021-82792-2 33568686 PMC7875995

[B36] McDonald J. C. Duffy D. C. Anderson J. R. Chiu D. T. Wu H. Schueller O. J. A. (2000). Fabrication of Microfluidic Systems in Poly(dimethylsiloxane). Electrophoresis 21 (1), 27–40. 10.1002/(SICI)1522-2683 10634468

[B37] Medrano J. V. Vilanova-Pérez T. Fornés-Ferrer V. Navarro-Gomezlechon A. Martínez-Triguero M. L. García S. (2018). Influence of Temperature, Serum, and Gonadotropin Supplementation in Short- and Long-Term Organotypic Culture of Human Immature Testicular Tissue. Fertil. Steril. 110 (6), 1045–1057. 10.1016/j.fertnstert.2018.07.018 30396549

[B38] Meurens F. Summerfield A. Nauwynck H. Saif L. Gerdts V. (2012). The Pig: a Model for Human Infectious Diseases. Trends Microbiol. 20 (1), 50–57. 10.1016/j.tim.2011.11.002 22153753 PMC7173122

[B39] Mulder R. L. Font-Gonzalez A. Hudson M. M. van Santen H. M. Loeffen E. A. H. Burns K. C. (2021). Fertility Preservation for Female Patients with Childhood, Adolescent, and Young Adult Cancer: Recommendations from the PanCareLIFE Consortium and the International Late Effects of Childhood Cancer Guideline Harmonization Group. Lancet Oncol. 22 (2), e45–e56. 10.1016/S1470-2045(20)30594-5 33539753

[B40] Oliver E. Stukenborg J. B. (2020). Rebuilding the Human Testis *In Vitro* . Andrology 8 (4), 825–834. 10.1111/andr.12710 31539453 PMC7496374

[B41] Perrard M.-H. Sereni N. Schluth-Bolard C. Blondet A. d'Estaing S. G. Plotton I. (2016). Complete Human and Rat *Ex Vivo* Spermatogenesis from Fresh or Frozen Testicular Tissue. Biol. Reproduction 95 (4), 89. 10.1095/biolreprod.116.142802 27580986

[B42] Place T. L. Domann F. E. Case A. J. (2017). Limitations of Oxygen Delivery to Cells in Culture: An Underappreciated Problem in Basic and Translational Research. Free Radic. Biol. Med. 113, 311–322. 10.1016/j.freeradbiomed.2017.10.003 29032224 PMC5699948

[B43] Portela J. M. D. de Winter-Korver C. M. van Daalen S. K. M. Meißner A. de Melker A. A. Repping S. (2019a). Assessment of Fresh and Cryopreserved Testicular Tissues from (Pre)pubertal Boys during Organ Culture as a Strategy for *In Vitro* Spermatogenesis. Hum. Reprod. 34 (12), 2443–2455. 10.1093/humrep/dez180 31858131 PMC6936721

[B44] Portela J. M. D. Mulder C. L. van Daalen S. K. M. de Winter-Korver C. M. Stukenborg J.-B. Repping S. (2019b). Strains Matter: Success of Murine *In Vitro* Spermatogenesis Is Dependent on Genetic Background. Dev. Biol. 456 (1), 25–30. 10.1016/j.ydbio.2019.08.007 31421080

[B45] Practice Committee of the American Society for Reproductive Medicine. Electronic address (2019). Fertility Preservation in Patients Undergoing Gonadotoxic Therapy or Gonadectomy: a Committee Opinion. Fertil. Steril. 112 (6), 1022–1033. 10.1016/j.fertnstert.2019.09.013 31843073

[B46] Price P. Goldsborough M. Tilkins M. (1998). Embryonic Stem Cell Serum Replacement International Patent Application. WO98/30679.

[B47] Reda A. Hou M. Winton T. R. Chapin R. E. Söder O. Stukenborg J.-B. (2016). In Vitrodifferentiation of Rat Spermatogonia into Round Spermatids in Tissue Culture. Mol. Hum. Reprod. 22 (9), 601–612. 10.1093/molehr/gaw047 27430551 PMC5013872

[B48] Richer G. Baert Y. Goossens E. (2020). In‐vitro Spermatogenesis through Testis Modelling: Toward the Generation of Testicular Organoids. Andrology 8 (4), 879–891. 10.1111/andr.12741 31823507 PMC7496450

[B49] Sato T. Katagiri K. Gohbara A. Inoue K. Ogonuki N. Ogura A. (2011). *In Vitro* production of Functional Sperm in Cultured Neonatal Mouse Testes. Nature 471 (7339), 504–507. 10.1038/nature09850 21430778

[B50] Saulnier J. Oblette A. Delessard M. Dumont L. Rives A. Rives N. (2021). Improving Freezing Protocols and Organotypic Culture: A Histological Study on Rat Prepubertal Testicular Tissue. Ann. Biomed. Eng. 49 (1), 203–218. 10.1007/s10439-020-02535-8 32440757

[B51] Schneider C. A. Rasband W. S. Eliceiri K. W. (2012). NIH Image to ImageJ: 25 Years of Image Analysis. Nat. Methods 9 (7), 671–675. 10.1038/nmeth.2089 22930834 PMC5554542

[B52] Sharma S. Klaverkamp R.-S. Wistuba J. Schlatt S. (2022). Limited Spermatogenic Differentiation of Testicular Tissue from Prepubertal Marmosets (*Callithrix jacchus*) in an *In Vitro* Organ Culture System. Mol. Cell. Endocrinol. 539, 111488. 10.1016/j.mce.2021.111488 34637880

[B53] Stukenborg J.-B. Schlatt S. Simoni M. Yeung C.-H. Elhija M. A. Luetjens C. M. (2009). New Horizons for *In Vitro* Spermatogenesis? an Update on Novel Three-Dimensional Culture Systems as Tools for Meiotic and Post-meiotic Differentiation of Testicular Germ Cells. Mol. Hum. Reprod. 15 (9), 521–529. 10.1093/molehr/gap052 19561342

[B54] Swindle M. Smith A. C. (1998). Comparative Anatomy and Physiology of the Pig. Scand. J. Laboratory Animal Sci. 25, 11–21.

[B55] Van Straaten H. W. M. Wensing C. J. G. (1978). Leydig Cell Development in the Testis of the Pig. Biol. Reprod. 18 (1), 86–93. 10.1095/biolreprod18.1.86 626770

[B56] Vermeulen M. Del Vento F. Kanbar M. Pyr Dit Ruys S. Vertommen D. Poels J. (2019). Generation of Organized Porcine Testicular Organoids in Solubilized Hydrogels from Decellularized Extracellular Matrix. Ijms 20 (21), 5476. 10.3390/ijms20215476 PMC6862040 31684200

[B57] Vollert I. Seiffert M. Bachmair J. Sander M. Eder A. Conradi L. (2014). *In-vitro* Perfusion of Engineered Heart Tissue through Endothelialized Channels. Tissue Eng. Part A 20 (3-4), 131025032956001–131025032956863. 10.1089/ten.TEA.2013.0214 24156346

[B58] Vreugdenhil P. K. Belzer F. O. Southard J. H. (1991). Effect of Cold Storage on Tissue and Cellular Glutathione. Cryobiology 28 (2), 143–149. 10.1016/0011-2240(91)90016-h 2070616

[B59] Wyns C. Kanbar M. Giudice M. G. Poels J. (2020). Fertility Preservation for Prepubertal Boys: Lessons Learned from the Past and Update on Remaining Challenges towards Clinical Translation. Hum. Reprod. Update 27, 433–459. 10.1093/humupd/dmaa050 33326572

[B60] Wyns C. Kanbar M. (2022). “ *In Vitro* Spermatogenesis,” in Female and Male Fertility Preservation. Editors Grynberg M. Patrizio P. (Cham: Springer International Publishing), 587–607. 10.1007/978-3-030-47767-7_44

[B61] Yamanaka H. Komeya M. Nakamura H. Sanjo H. Sato T. Yao M. (2018). A Monolayer Microfluidic Device Supporting Mouse Spermatogenesis with Improved Visibility. Biochem. Biophysical Res. Commun. 500 (4), 885–891. 10.1016/j.bbrc.2018.04.180 29705697

[B62] Yokonishi T. Sato T. Komeya M. Katagiri K. Kubota Y. Nakabayashi K. (2014). Offspring Production with Sperm Grown *In Vitro* from Cryopreserved Testis Tissues. Nat. Commun. 5, 4320. 10.1038/ncomms5320 24984101

