# Supplementary material for: Microfluidic and Static Organotypic Culture Systems to Support Ex Vivo Spermatogenesis From Prepubertal Porcine Testicular Tissue: A Comparative Study
Source: Front Physiol. 2022 Jun 2;13:884122. doi: 10.3389/fphys.2022.884122 (PMC9201455; doi:10.3389/fphys.2022.884122)
Supplement: Supplementary file 1 [file DataSheet1.pdf]

Supplementary Material

1 Supplementary Tables

Supplementary Table 1: Primary antibodies used for the experiments

Antibody	Target	Dilution	Supplier + Reference
PGP9.5	Undifferentiated spermatogonia	1:2000	Dako – Z5116
VASA	Germ cells	1:2000	Abcam – ab13840
SYCP3	Spermatocytes	1:2000	Sigma – HPA039635
CREM	Round spermatids	1:500	Santa Cruz – SC101530
Ki67	Proliferation marker	1:500	Dako – M7240
SOX9	Sertoli cells	1:3000	Abcam – ab185966
INSL3	Leydig cells	1:2000	Sigma – A96525
MDA	Lipid peroxidation end-product	1:2000	Abcam – ab6463
Cleaved caspase-3	Apoptotic cells	1:250	Cell Signaling - #9661S

2 Supplementary Figures

Supplementary Figure 1. MF and PDMS cover chips design and fabrication

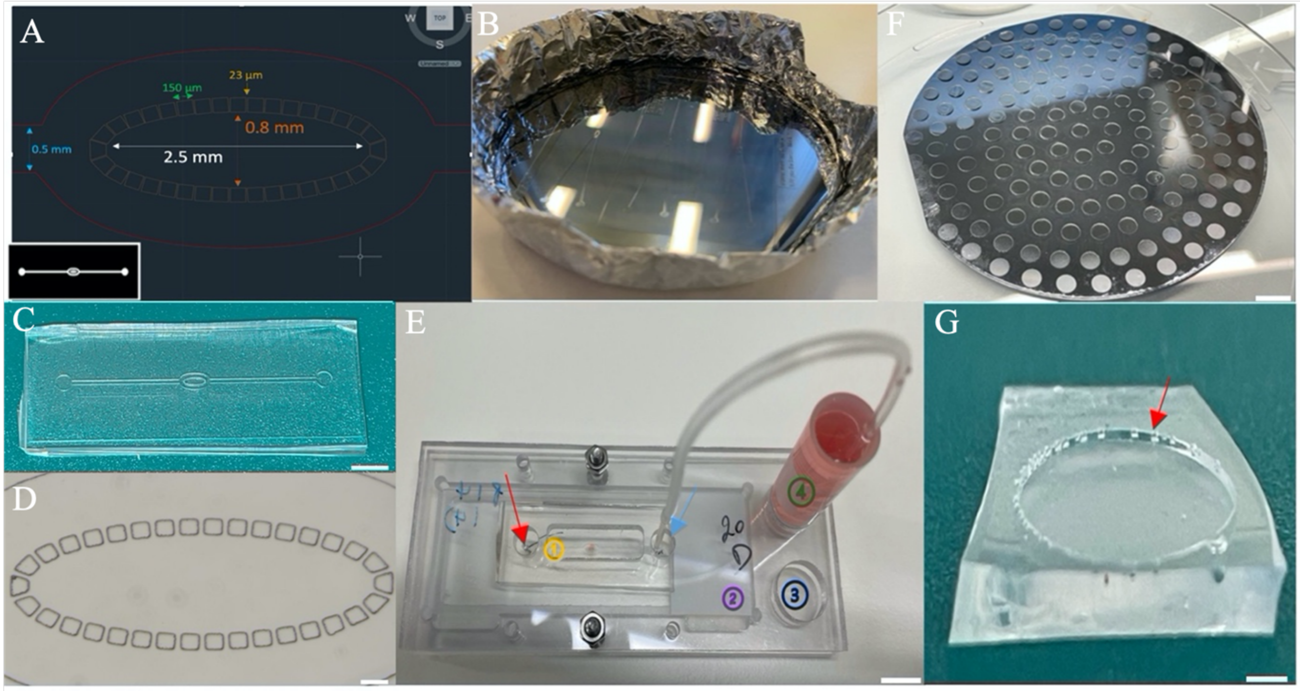

(A): Design of the MF chip. Channel width (0.5 mm), chamber (length: 2.5 mm, width: 0.8 mm), pillars (length: 0.15 mm, width: 0.15 mm), interpillar-distance (23 µm), and chamber depth (≃170 µm). (B): Replication of the MF chip by molding of the PDMS over the patterned silica mold. (C): individual patterned MF chip after cutting. Scale bar is 5 mm. (D): MF chamber with pillars in patterned PDMS, shown at 400x magnification. Scale bar is 0.2 mm. (E): Final MF chip: the PDMS layer (1), microscope slide (2) tailored plastic support (3) culture media reservoir (4) Blue arrow: inlet, red arrow: outlet, to be connected to the syringe-pump. Scale bar 10 mm. (F): Patterned silica mold for PDMS cover chips. Scale bar 10 mm. G: final PDMS cover chip. Scale bar 1 mm.

Supplementary Figure 2. Scores used for Histology and Immunohistochemistry semi-quantitative analyses

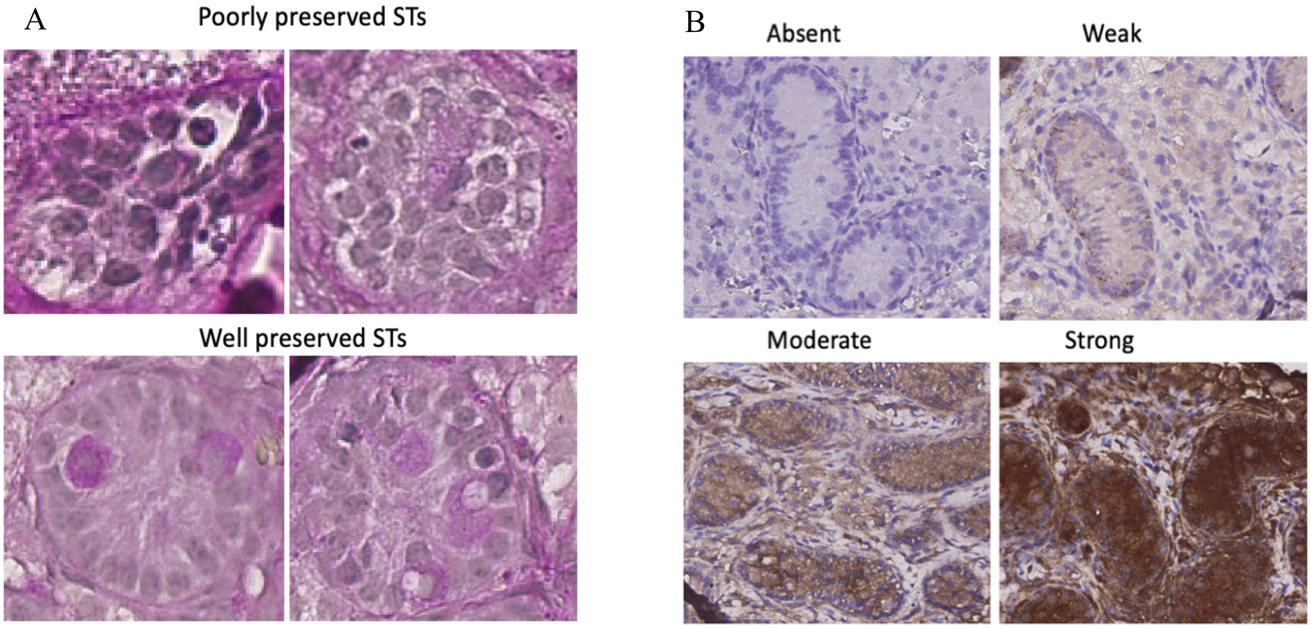

(A) Seminiferous integrity score. (B) Intensity score for MDA analysis.

Supplementary Figure 3. Duplex immunofluorescence for germ cell differentiation

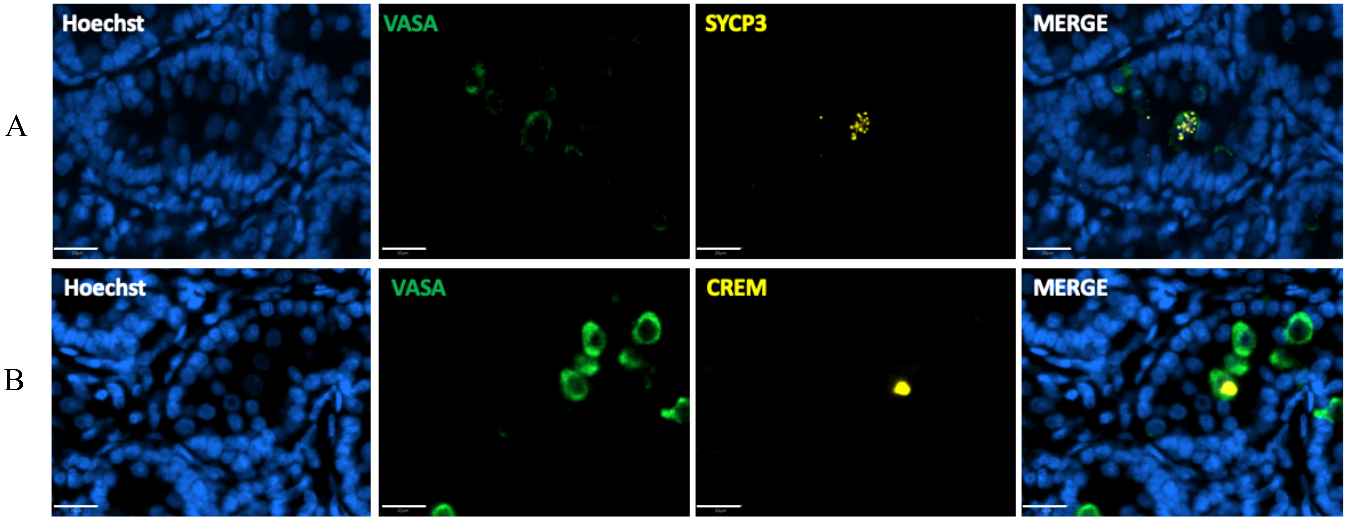

Staining for VASA/SYCP3 (A) and VASA/CREM (B) confirmed the germ cell identification and localization of SYCP3 and CREM. Scale bars = 20 µm.

Supplementary Figure 4. Immunohistochemistry controls

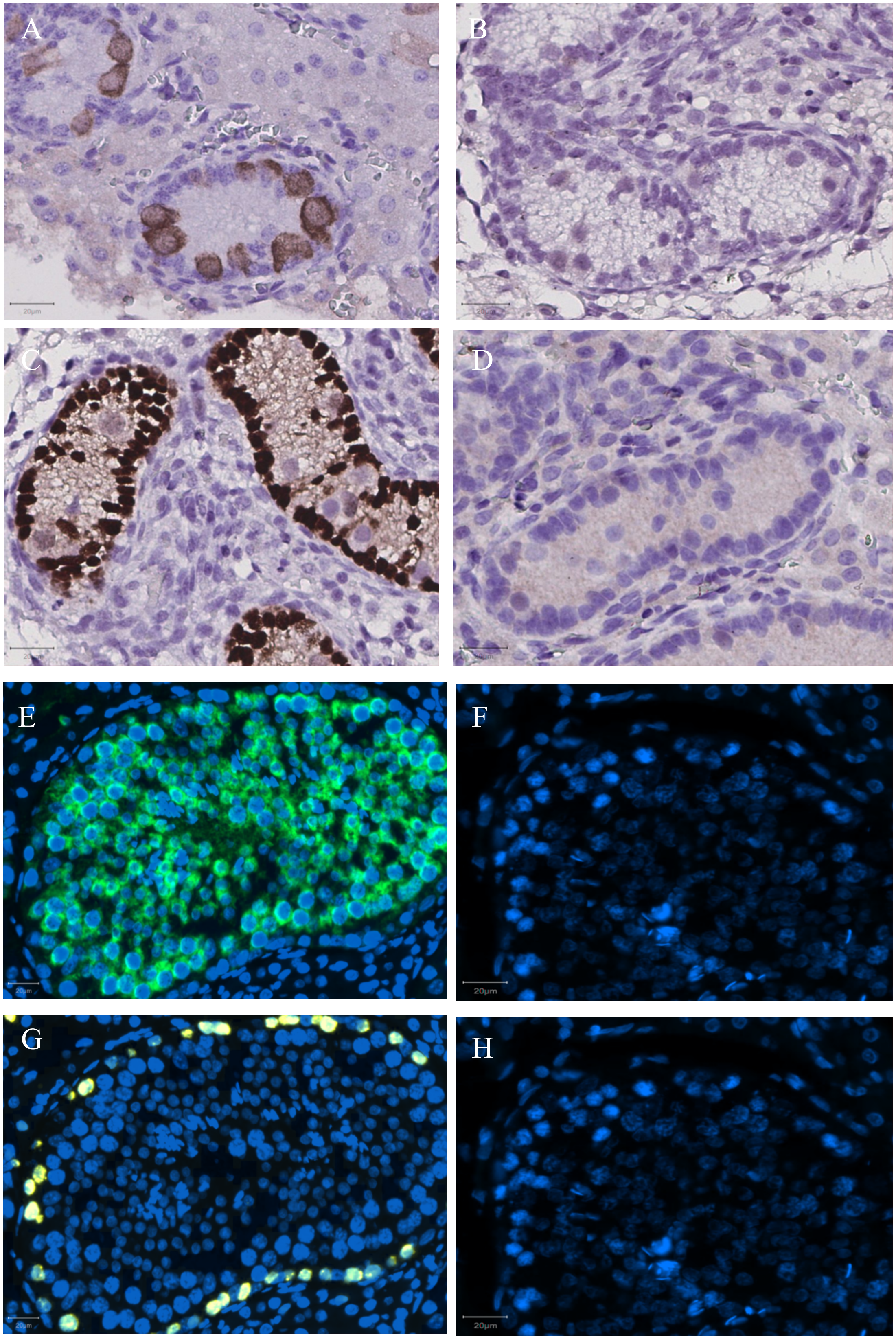

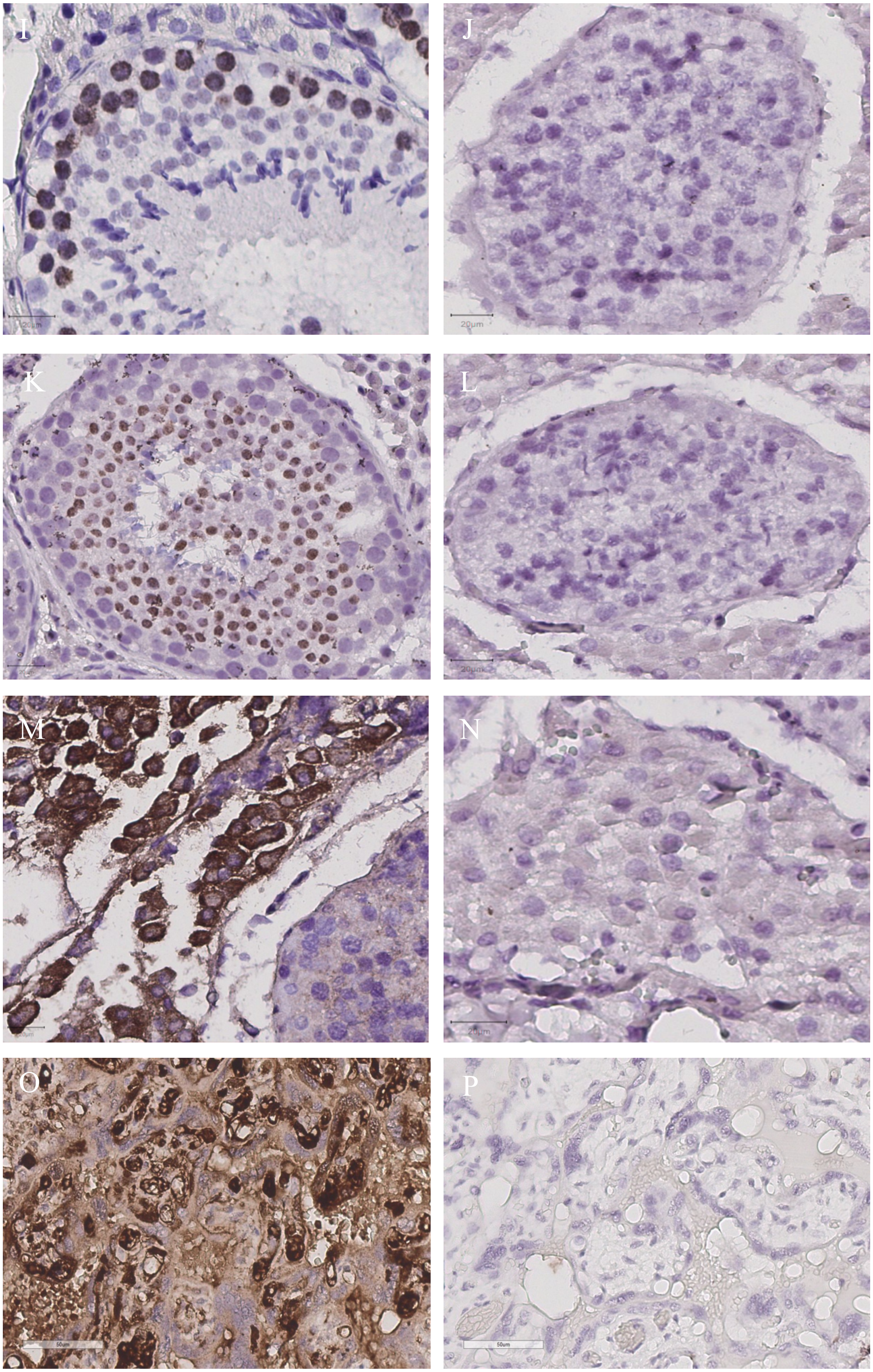
I
J
20um
K
L
20um
M
N
O
50um
P
50um

Tissues incubated with the primary antibody were used as positive controls and those without the primary antibody but with: antibody diluent, secondary antibody and detections reagents, were used as a negative control.

Porcine ITT recovered from pigs aged between 4 and 7 days old was used as positive and negative control for PGP9 (A, B), SOX9 (C, D) immunostainings. Porcine mature testicular tissue (6 months old) was used as positive and negative control for VASA (E, F), Ki67 (G, H), SYCP3 (I, J), CREM (K, L) and INSL3 (M, N). Third trimester placental tissue was used as a positive and negative control for MDA (O, P). Scale bars are shown in the lower left quadrant of each image (Scale bars in A to N are equal to 20 μm and in O and P to 50 μm).

Supplementary Figure 5. Graphical results for the comparison among the four systems (and not to control) using Tukey's multiple pairwise comparison test

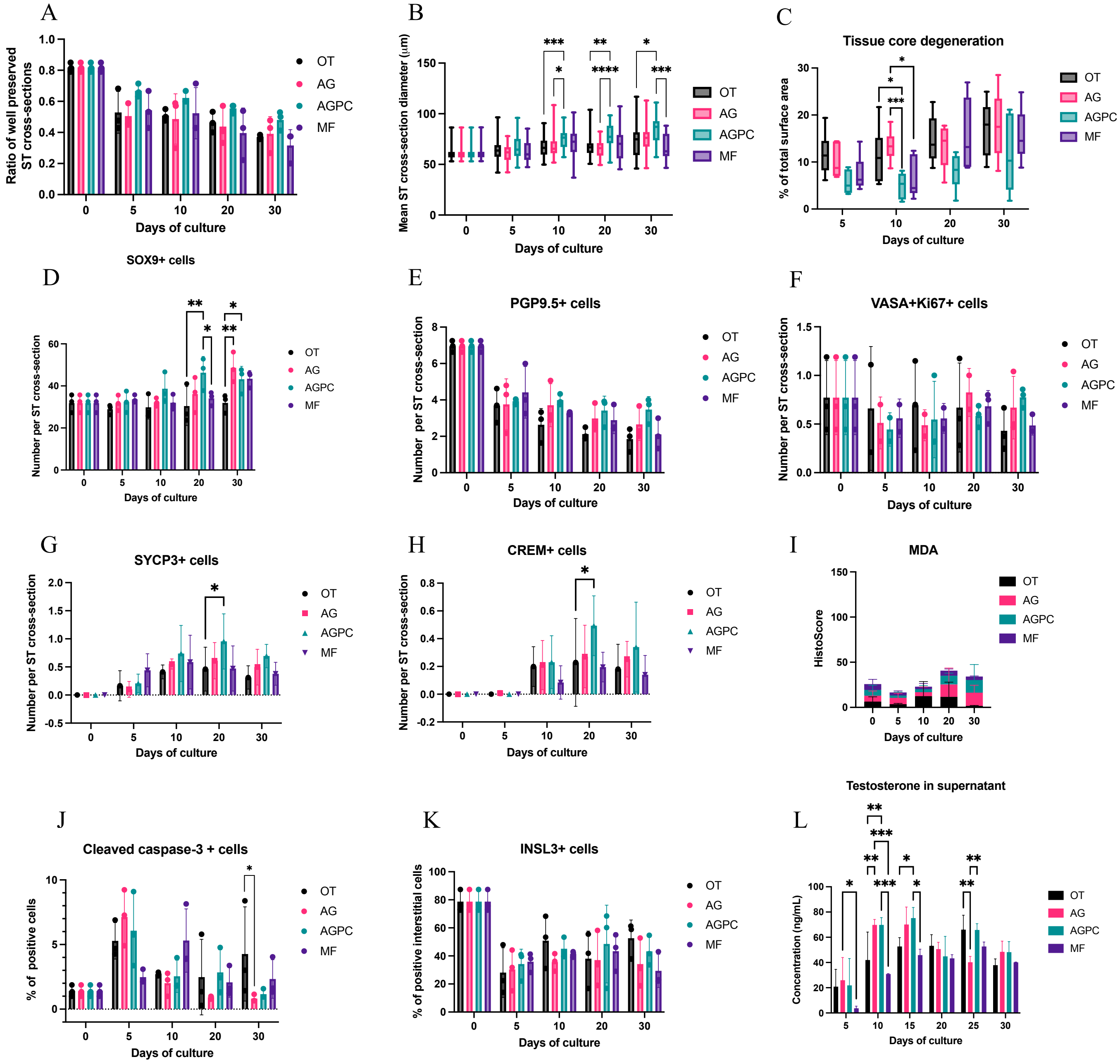

A: Ratio of well-preserved ST cross-sections, B: Mean ST cross-section diameters, C: Percentage of tissue surface with core degeneration, D: Number of SOX9+ cells/ST cross-section, E: Number of PGP9.5+ cells/ST cross-section, F: Number of VASA+Ki67+ cells/ST cross-section, G: Number of SYCP3+ cells/ST cross-section, H: Number of CREM+ cells/ST cross-section, I: HistoScore for MDA, J: Percentage of cleaved-caspase3+ cells among total testicular cells, K: Percentage of INSL3+ cells among total interstitial cells, L: Testosterone concentrations (ng/mL).
